# Gas-Phase Reactions of Dimethyl Disulfide with Aliphatic Carbanions - A Mass Spectrometry and Computational Study

**DOI:** 10.1007/s13361-017-1858-x

**Published:** 2018-01-08

**Authors:** Barbara Franczuk, Witold Danikiewicz

**Affiliations:** Institute of Organic Chemistry, Polish Academy of Sciences, Kasprzaka 44/52, 01-224, Warsaw, Poland

**Keywords:** Gas-phase ion-molecule reactions, Thiophilic reaction, Dimethyl disulfide, Aliphatic carbanions, DFT calculations

## Abstract

Ion-molecule reactions of Me_2_S_2_ with a wide range of aliphatic carbanions differing by structure and proton affinity values have been studied in the gas phase using mass spectrometry techniques and DFT calculations. The analysis of the spectra shows a variety of product ions formed via different reaction mechanisms, depending on the structure and proton affinity of the carbanion. Product ions of thiophilic reaction (*m/z* 47), S_N_2 (*m/z* 79), and E2 elimination – addition sequence of reactions (*m/z* 93) can be observed. Primary products of thiophilic reaction can undergo subsequent S_N_2 and proton transfer reactions. Gibbs free energy profiles calculated for experimentally observed reactions using PBE0/6-311+G(2d,p) method show good agreement with experimental results.

**Electronic supplementary material:**

The online version of this article (10.1007/s13361-017-1858-x) contains supplementary material, which is available to authorized users.

**Graphical Abstract:**
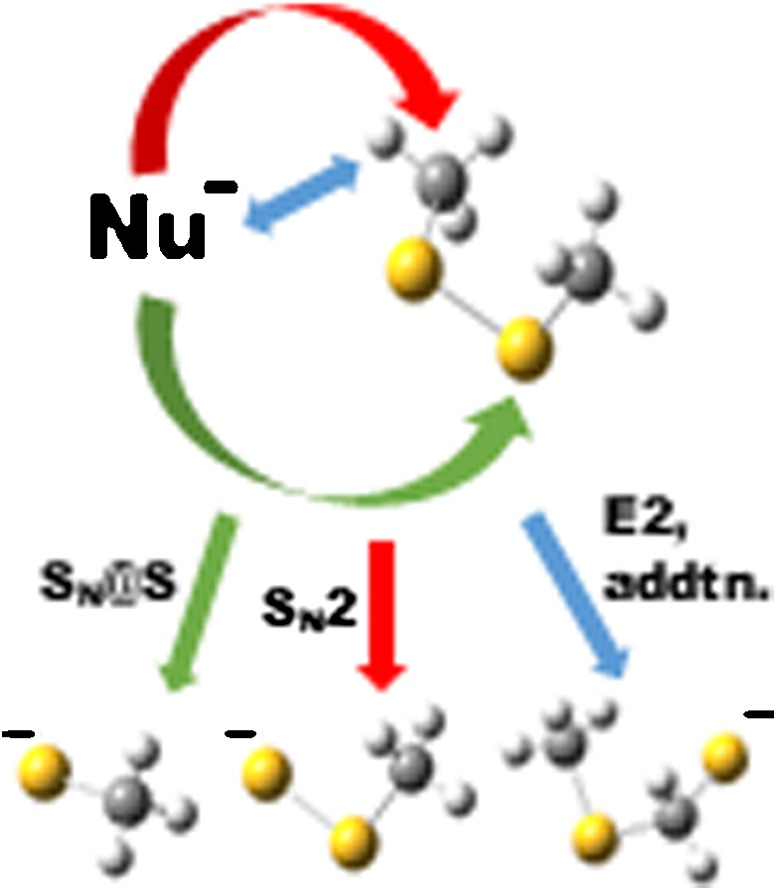
ᅟ

## Introduction

Disulfides, due to their importance in biochemistry and atmospheric processes, have been a subject of many experimental [1, 2] and theoretical [3–12] studies. The results of these studies were summarized in two reviews [13, 14].

In biochemistry, S–S bonds enable correct protein folding and stabilize protein structure [15, 16]. They also work as redox switches that protect the thiol groups from over-oxidation and affect gene expression [17, 18]. Mechanism of thiolate-disulfide exchange has been a subject of considerable investigation [4–8]. Experimental studies suggest that in liquid phase S_N_2 reaction occurs, however according to theoretical calculations performed by Bachrach and coworkers [4, 5] for disulfides with small substituents on the sulfur atoms, reaction proceeds through a two-step addition-elimination mechanism. Computations show that larger substituents on sulfur atom prevent stable intermediate from forming and reaction proceeds through S_N_2 pathway. Thiolate-disulfide exchange was also examined using chemical dynamics simulations for reactions of dimethyl disulfide and hydrogen disulfide [7]. These results also indicate that the main reaction pathway is an addition-elimination process.

Dimethyl disulfide is one of the sulfur compounds released to the troposphere by biogenic sources [19]. Me_2_S_2_ is highly reactive in the gas phase and its reactions with some atmospheric oxidants such as ·OH [11] and hydrated radical anions [10] have been studied.

In the liquid phase [14], the most characteristic reaction involving disulfides and nucleophiles is cleavage of the S–S bond according to the nucleophilic substitution at the sulfur atom mechanism (thiophilic reaction, S_N_@S).

The thiophilic reactions, which have been observed for dialkyl disulfides in the liquid phase and can take place also in the gas phase, have been studied by Grabowski and Zhang [1]. Using the flowing afterglow-mass spectrometry (FA-MS) method, these authors have studied the reactions of dimethyl disulfide with a number of small nucleophiles with different proton affinity (PA) values. For very strong bases like NH_2_^-^, OH^-^, CH_3_O^-^, and PhCH_2_^-^ ions, two ionic products were formed predominantly or exclusively: CH_3_S^-^ (*m/z* 47) and *m/z* 93 ions. At first glance the *m/z* 93 ion should have the structure of deprotonated dimethyl disulfide: CH_3_-S-S-CH_2_^-^. However, Grabowski and Zhang gave convincing evidence that the real structure of this ion corresponds to thioformaldehyde methyl hemithioacetal anion: CH_3_-S-CH_2_-S^-^. In the reactions with anions with lower PA values and containing carbanion stabilizing group, products formed by deprotonation of the primary neutral product of the thiophilic reaction have been observed, for example:

$$ {}^{-}{\mathrm{CH}}_2{\mathrm{NO}}_2+{\mathrm{CH}}_3-\mathrm{S}-\mathrm{S}-{\mathrm{CH}}_3\to {\mathrm{CH}}_3-\mathrm{S}-{\mathrm{CH}}_2{\mathrm{NO}}_2+{\mathrm{CH}}_3{\mathrm{S}}^{-}\to {\mathrm{CH}}_3-\mathrm{S}-{\mathrm{CH}}^{\left(-\right)}-{\mathrm{NO}}_2+{\mathrm{CH}}_3\mathrm{SH} $$

Additionally, Bachrach and Pereverzev conducted theoretical calculations for gas-phase reaction of methyl disulfide and dimethyl disulfide with F^-^, OH^-^ and allyl anion [6]. The goal of these studies was to determine the mechanism of the occurring elimination and substitution reactions. Calculations that were carried out suggest that CH_3_-S-CH_2_-S^-^ ion is formed by recombination of the products of E2-type elimination reaction (CH_3_S^-^ and CH_2_=S, Scheme 2).

**Scheme 1:**
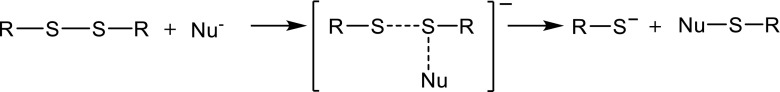
S_N_@S reaction of dialkyl disulfides with nucleophiles

**Scheme 2:**
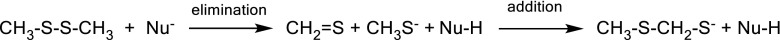
Formation of CH_3_-S-CH_2_-S^-^ ion

Continuing our research in the field of gas-phase reactions of carbanions with electrophiles, we decided to study in more detail the reactions of dimethyl disulfide with a wide range of aliphatic carbanions differing by structure and proton affinity values. An important novel part of our work is extensive DFT calculations used to rationalize the experimental results.

## Experimental

All reagents and solvents used in this work are commercially available and were used without additional purification.

The experiments were performed using a modified API 3000 (AB Sciex, Framingham, MA, USA) triple quadrupole mass spectrometer equipped with TurboIonSpray electrospray ionization (ESI) ion source. A majority of carbanions were generated in the ESI source by decarboxylation of the respective carboxylic acids anions [20, 21]. This method could not be used for generation of acetonyl enolate anion due to high instability of acetoacetic acid. Acetonyl anion can be, however, generated efficiently from the anion of ethyl acetoacetate: an attempt to generate ethyl acetate enolate anion by decarboxylation of monoethyl malonate anion gave not only the expected *m/z* 87 ion but also *m/z* 59 and 41 ions, to which the structures of acetic acid enolate anion and ketene anion have been assigned (see discussion in the following chapter). All three ions were used in the reactions with Me_2_S_2_. Methyl and ethyl prop-2-ynoate anions (^-^C≡C-CO_2_Me and ^-^C≡C-CO_2_Et ) were generated from the monoesters of acetylenedicarboxylic acid obtained by partial hydrolysis with NaOH of the respective diesters in a vial, followed by decarboxylation in the ion source. Nitromethyl anion (^–^CH_2_NO_2_) was generated by deprotonation of nitromethane with sodium methoxide.

Dimethyl disulfide was delivered as a vapor with nitrogen used as a collision gas to the collision cell where the reactions take place. Source parameters and the voltages for the entrance ion optics were optimized for maximum production and transmission of the carbanions of interest. Collision energy was set as low as possible (–1 eV) for all examined carbanions to prevent ion fragmentation. Carboxylic acids were dissolved in methanol (HPLC grade) and injected with a syringe pump at a flow rate of 20 μL min^-1^. Data were processed using Analyst 1.4 program (AB Sciex). In API 3000 mass spectrometer it is not possible to estimate the concentration of the reagent vapors in a collision gas, so all experiments have only qualitative character.

All calculations were performed with Gaussian 09 Revision D.01 program [22]. The Cartesian coordinates of initial geometries were created using GaussView program [23]. Density functional theory (DFT) calculations were performed at PBE1PBE/6-311+G(2d,p) level of theory for geometry optimization, calculation of vibrational frequencies, and calculation of the enthalpy and Gibbs free energy (PBE1PBE is a Gaussian notation for PBE0). All calculations were performed at the standard conditions (298.15 K and 1 bar). It was checked that all stable structures had only real vibrational frequencies and the transition states have one imaginary frequency. Transition state structures have been found on the Potential Energy Surface after scan along the reaction pathway using standard Berny algorithm (Gaussian optimization keyword *opt=TS*). Our experience shows that the 6-311+G(2d,p) basis set gives good accuracy for the geometries, frequencies, and energies of the small- and medium-sized organic anions and neutral molecules [24–26]. For additional verification of the accuracy of the selected DFT method, calculations were performed for selected reactions using G4(MP2) composite method, which was designed to give most accurate thermochemical parameters using acceptable computer resources [27].

## Results and Discussion

**Figure 1:**
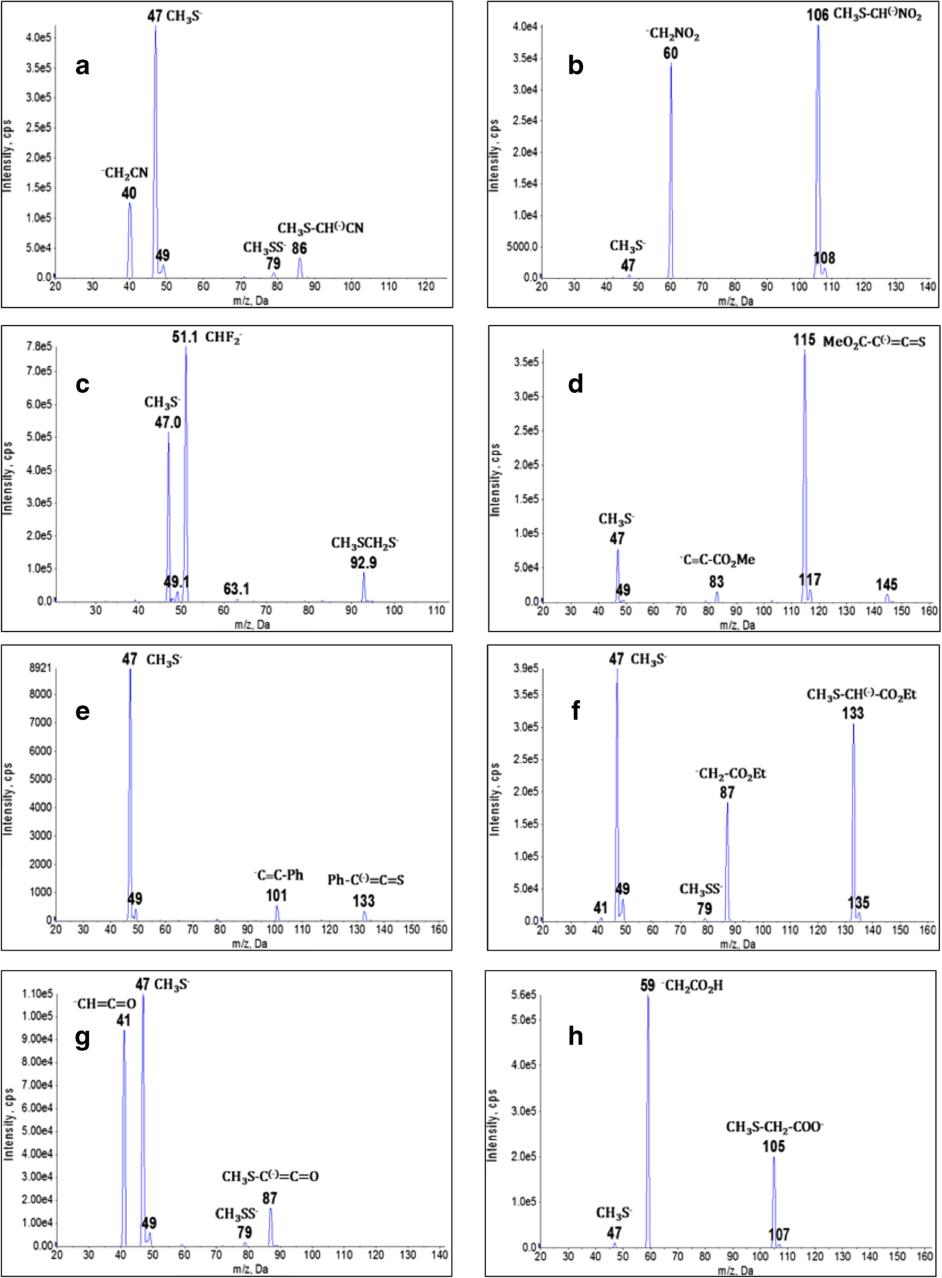
Mass spectra of the reaction products of Me_2_S_2_ with: **(a)** ^–^CH_2_-CN, **(b)** ^–^CH_2_NO_2_, **(c)** CHF_2_^-^, **(d)** ^–^C≡C-CO_2_Me, **(e)** ^–^C≡C-Ph, **(f)** ^–^CH_2_-COOEt, **(g)** ^–^CH=C=O, **(h)** ^–^CH_2_COOH

**Scheme 3:**
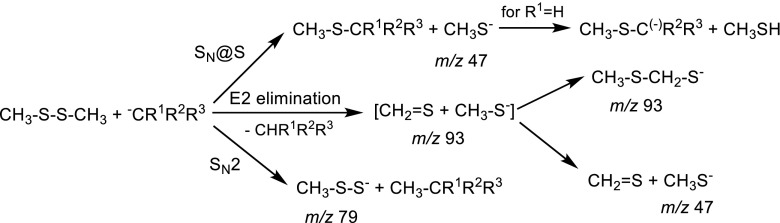
Possible reactions between Me_2_S_2_ and primary or secondary carbanions in the gas phase

For the studies of the reactions of aliphatic carbanions with dimethyl disulfide in the gas phase we selected six primary anions: nitromethyl, acetonyl, (methoxycarbonyl)methyl, (ethoxycarbonyl)methyl, carboxymethyl (i.e., acetic acid enolate anion – see discussion below), and cyanomethyl, three secondary anions: difluoromethyl, dichloromethyl, and ketene anion and five tertiary anions: trifluoromethyl, trichloromethyl, phenylacetylenide, (methoxycarbonyl)acetylenide, and (ethoxycarbonyl)acetylenide. Table 1 summarizes semiquantitative results of these reactions. Spectra recorded for eight representative reactions are presented in Figure 1 (for others see Supporting Information). All possible reactions between dimethyl disulfide and aliphatic carbanions in the gas phase are collected in Scheme 3.

**Table 1:** Semiquantitative Results of the Reactions of Me_2_S_2_ with Aliphatic Carbanions

No.	Carbanion (*m/z*) rel. int.	PA [kcal mol^-1^]	Relative intensity			CH_3_S-C^(-)^R_1_R_2_ (*m/z*) rel. int.	other products (*m/z*) rel. int.
			CH_3_S^-^(*m/z* 47)	CH_3_S-S^-^(*m/z* 79)	CH_3_SCH_2_S^-^(*m/z* 93)		
1.	^–^CH_2_NO_2_ (60) 84%	357	1.1%	-	-	CH_3_S-CH^(-)^-NO_2_ (106) 100%	-
2.	CCl_3_^-^ (117) 100%	358	1.3%	-	-	-	-
3.	^–^C≡C-CO_2_Me (83) 4.3%	359	21.5%	-	-	-	MeO_2_C-C^(-)^=C=S (115) 100%
4.	^–^C≡C-CO_2_Et (97) 100%	359a	1.4%	-	-	-	EtO_2_C-C^(-)^=C=S (129) 46%
5.	^–^CH=C=O (41) 85%	365	100%	1.6%	-	CH_3_S-C^(-)^=C=O (87) 15%	-
6.	^–^CH_2_CO_2_H (59) 100%	368	2.1%	-	-	CH_3_S-CH_2_-COO^-^ (105) 36%	-
7.	CH_3_-CO-CH_2_^-^ (57) 27%	369	100%	-	-	CH_3_SCH^(-)^-CO-CH_3_ (103) 67%	-
8.	^–^CH_2_-CO_2_Et (87) 47%	369	100%	1.1%	-	CH_3_S-CH^(-)^-CO_2_Et (133) 78%	-
9.	^-^C≡C-Ph (101) 6.5%	371	100%	-	-	-	Ph-C^(-)^=C=S (133) 4.3%
10.	^–^CH_2_-CO_2_Me (73) 10.2%	372	100%	-	-	CH_3_S-CH^(-)^-CO_2_Me (119) 17.2%	(CH_3_S)_2_-C^(-)^-CO_2_Me (165) 8%
11.	^–^CH_2_-CN (40) 41%	372	100%	2.2%	-	CH_3_S-CH^(-)^-CN (86) 8.1%	-
12.	CHCl_2_^–^ (83) 34%	375	100%	-	5.4%	CH_3_S-CCl_2_^-^ (129) 11%	-
13.	CF_3_^–^(69) 12%	378	100%	2%	4.3%	-	CF_3_S-(101) 10.7%
14.	CHF_2_^–^ (51) 100%	389	66%	-	12%	-	-

The reactions are ordered according to increasing proton affinity of the carbanion

^a^Because there was no experimental PA data for this anion, calculations using advanced G4(MP2) compound method have been performed for methyl and ethyl esters of acetylenecarboxylic acid. The results were 361.1 and 361.4 kcal mol^-^1, respectively, so we decided to assign for the anion of ethyl acetylenecarboxylate the same PA value as for the methyl ester.

### Reactions of Cyanomethyl Anion

**Scheme 4:**
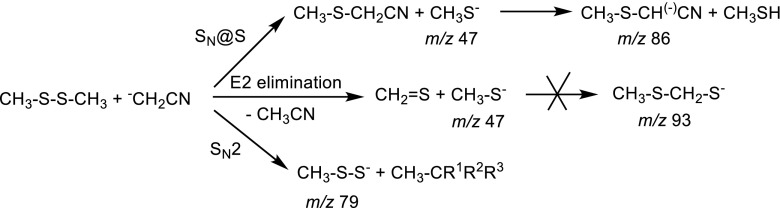
Reactions between Me_2_S_2_ and cyanomethyl anion in the gas phase

Cyanomethyl anion can serve as the representative example of the studied primary carbanions with medium proton affinity value (PA = 372 kcal mol^-1^). The main peak in the spectrum of the reaction products comes from the CH_3_S^-^ ion (*m/z* 47). It can be formed either in the thiophilic reaction (S_N_@S) or in E2 elimination process (Scheme 4).

The presence of the *m/z* 86 peak corresponding to the anion of methylthioacetonitrile confirms that the S_N_@S reaction really takes place. The yield of this reaction is very low because the proton affinity of the methythio anion is comparable to that of the anion of methylthioacetonitrile. Experimental values (NIST Chemistry Webbook [28]) are identical within the experimental error (357.6 ± 2.5 kcal mol^-1^) but DFT calculations by PBE1PBE/6-311+G(2d,p) method show that CH_3_S^-^ ion is slightly more basic (357.5 versus 355.2 kcal mol^-1^). More accurate G4(MP2) calculations also show that CH_3_S^-^ ion is more basic (358.5 versus 358.1 kcal mol^-1^) but the difference is marginal. It has to be noted, however, that Gibbs free energy change in the reaction:

$$ {\mathrm{CH}}_3\mathrm{S}-{\mathrm{CH}}_2-\mathrm{CN}+{\mathrm{CH}}_3{\mathrm{S}}^{-}\to {\mathrm{CH}}_3\mathrm{S}-{\mathrm{CH}}^{\left(-\right)}-\mathrm{CN}+{\mathrm{CH}}_3\mathrm{S}\mathrm{H} $$

is higher than the change in enthalpy (–3.7 kcal mol^-1^ by PBE0 method and –1.7 kcal mol^-1^ by G4(MP2) method) so the reaction equilibrium should be shifted completely to the products. The relatively low abundance of the CH_3_S-CH^(-)^-CN ion is most likely the result of relatively low reaction rate together with the short dwell time of the ions in a collision chamber of the triple quadrupole mass spectrometer.

The second reaction product that can be observed in the spectrum, but in a very low abundance, is *m/z* 79 ion corresponding to CH_3_S-S^-^ ion, which results from the S_N_2 reaction. The third possible process: the elimination-addition reaction does not take place for these substrates because the *m/z* 93 peak corresponding to the CH_3_-S-CH_2_-S^-^ ion is not observed in the spectrum. This result, however, does not exclude that the first step, i.e., E2 elimination can proceed, yielding methylthio anion (Scheme 4).

**Figure 2:**
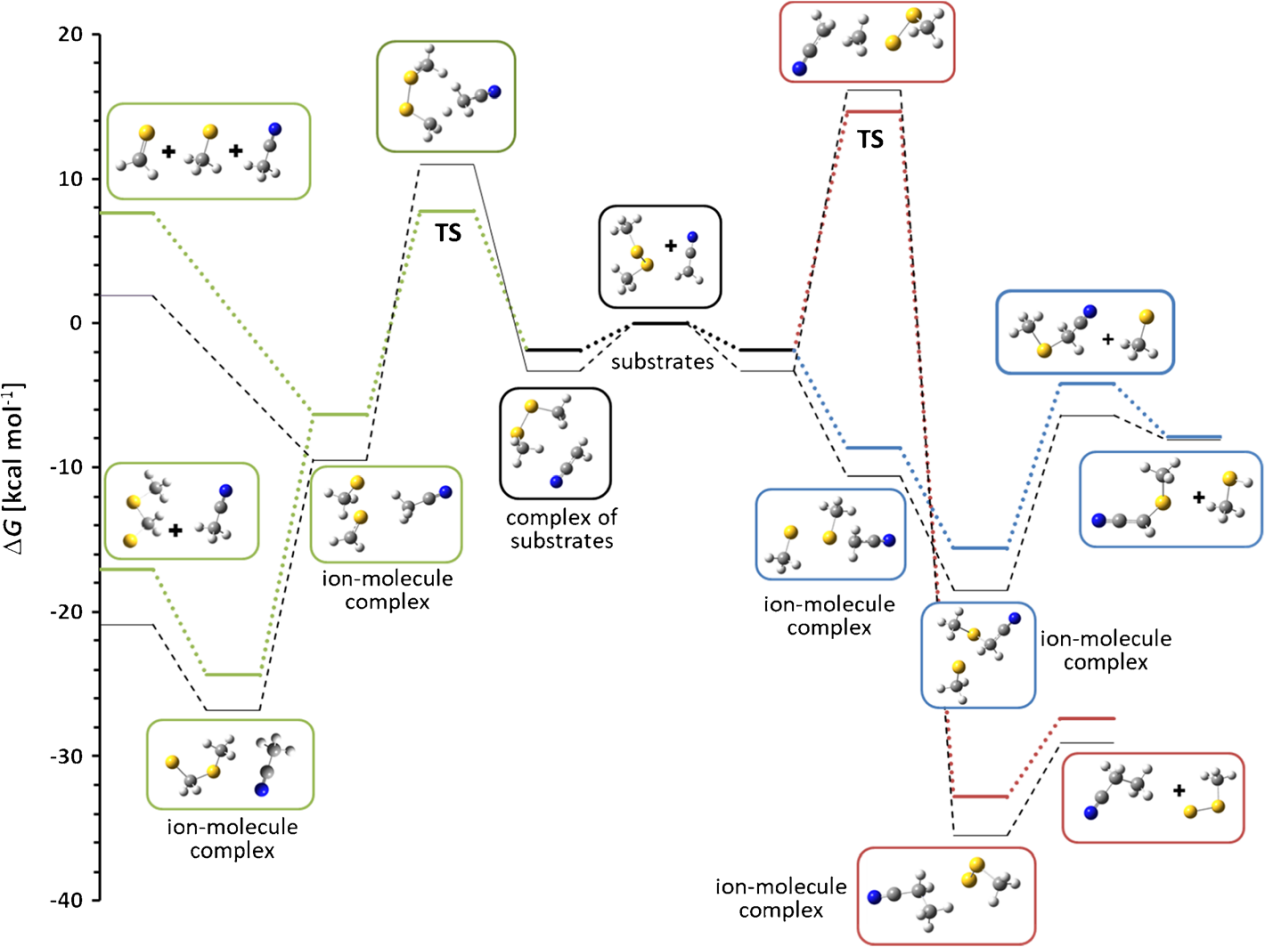
Calculated Gibbs free energy diagram of possible paths of the reaction between cyanomethyl anion and dimethyl disulfide in the gas phase. Blue trace: thiophilic reaction, red trace: S_N_2 reaction, green trace: elimination – addition reaction, thin black line: the results of G4(MP2) calculations

Calculated Gibbs free energy profiles of these reactions are in agreement with the experimental results. According to our calculations, thiophilic reaction is an exoergic process, which proceeds without an activation barrier. It is possible to find a local minimum corresponding to ion-molecule complex of substrates (Figure 2) but a different orientation of the reacting species leads directly to ion-molecule complex of CH_3_S^-^ ion with methylthioacetonitrile molecule (Δ*G* = –8.7 kcal mol^-1^). It has to be noted that the C–S bond in the latter is slightly longer than in a free molecule of this species (1.93 versus 1.83 Å). In the next step, this complex is transformed into another one comprised of the same species but with different geometry and significantly lower energy (Δ*G* = –15.6 kcal mol^-1^). This complex can either dissociate to CH_3_S^-^ ion and methylthioacetonitrile molecule or subsequent proton transfer reaction can occur, yielding methanethiol and the anion of methylthioacetonitrile.

S_N_2 reaction is highly exoergic (∆*G* of the complex of products = –32.8 kcal mol^-1^), but it has high activation free energy (14.7 kcal mol^-1^), explaining very low intensity of the product peak in mass spectrum. The results of the calculations do not exclude the E2 elimination process because its ∆*G*^≠^ is relatively low and, simultaneously, the ion-molecule complex of the E2 elimination products has enough energy to decompose to separated CH_3_S^-^, CH_2_=S, and CH_3_CN molecules. This can prevent the subsequent addition reaction, so the only observed ionic product is methylthio anion (*m/z* 47).

However, our previous experience shows that PBE0 (PBE1PBE in Gaussian) DFT method with relatively large 6-311+G(2d,p) basis set reproduces quite well thermochemistry of typical organic reactions, so we decided to repeat the calculations for the reactions of dimethyl disulfide with cyanomethyl anion using G4(MP2) composite method, which was designed to give accurate thermochemical results for small- and medium-sized organic molecules and ions [27]. The results (Figure 2) showed that G4(MP2) method gives Δ*G* values, which are close to those obtained by PBE0 method with 6-311+G(2d,p) basis set. Interestingly, G4(MP2) method gives higher activation barriers comparing to PBE0 and lower free energies for the reaction products and their ion-molecule complexes. These differences, however, do not change the general picture of the observed reactions. These results are very important from the point of view of our further research, in which much larger molecules will be studied. For such molecules, G4(MP2) method is impractical due to very long computation time, even on advanced workstations.

### Reactions of Nitromethyl Anion

Nitromethyl anion possesses the weakest basic properties among carbanions studied within this work (PA = 357 kcal mol^-1^). Despite this it reacts efficiently with dimethyl disulfide in the gas phase according to the thiophilic reaction mechanism yielding *m/z* 106 ion:

$$ {\mathrm{CH}}_3-\mathrm{S}-\mathrm{S}-{\mathrm{CH}}_3+{}^{-}{\mathrm{CH}}_2{\mathrm{NO}}_2\to {\mathrm{CH}}_3{\mathrm{S}}^{-}+{\mathrm{CH}}_3-\mathrm{S}-{\mathrm{CH}}_2{\mathrm{NO}}_2\to {\mathrm{CH}}_3\mathrm{SH}+{\mathrm{CH}}_3-\mathrm{S}-{\mathrm{CH}}^{\left(-\right)}-{\mathrm{NO}}_2 $$

**Figure 3:**
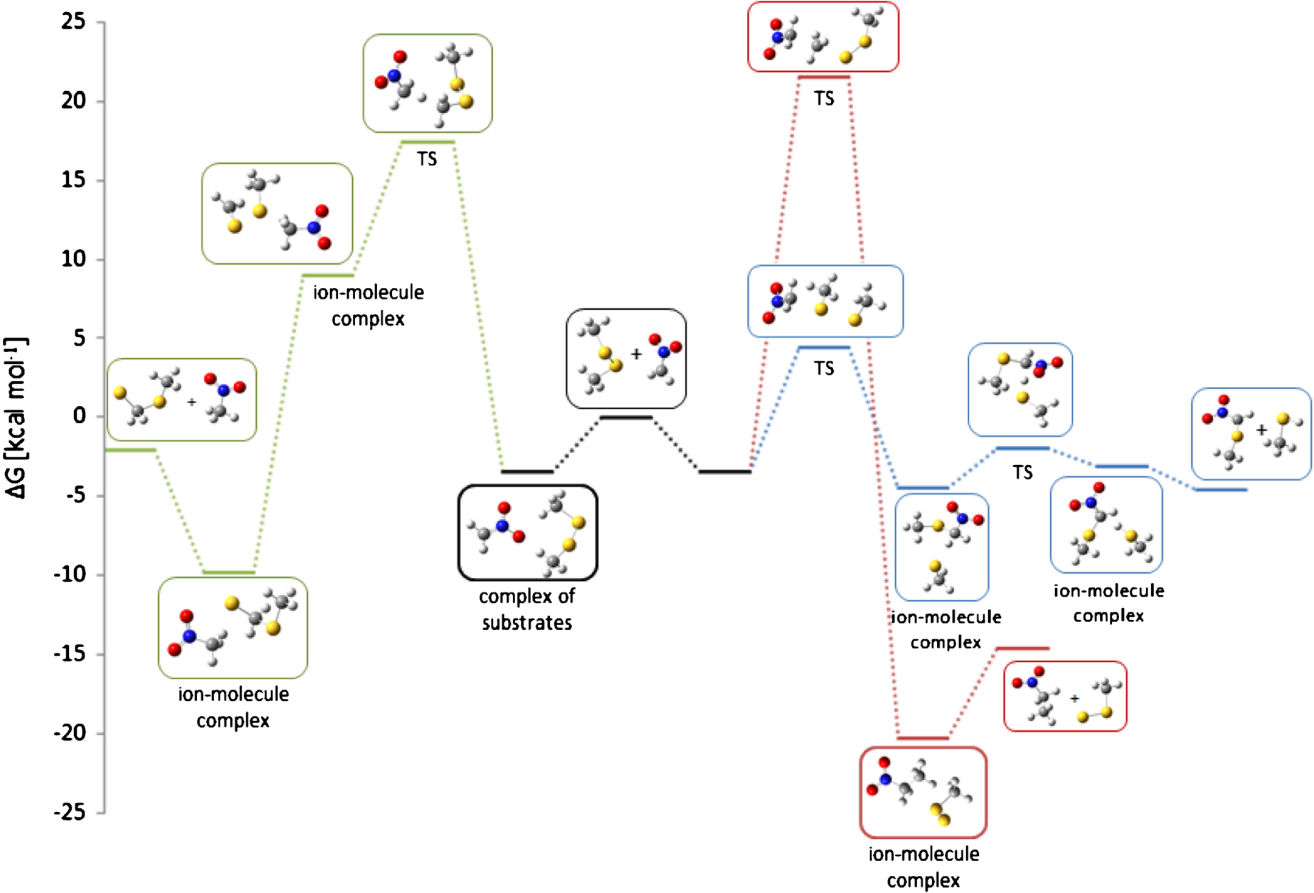
Calculated Gibbs free energy diagram of possible paths of the reaction between nitromethyl anion and dimethyl disulfide in the gas phase. Blue trace: thiophilic reaction, red trace: S_N_2 reaction, green trace: elimination – addition reaction

No other product ions, except of the traces of methylthio anion (*m/z* 47), are observed. This result can be easily rationalized by modeling of the possible reaction pathways (Figure 3). The thiophilic reaction (blue line) requires very low activation free energy, i.e., about 4.5 kcal mol^-1^. The Δ*G*^≠^ of the S_N_2 reaction (red line) is much higher (21.5 kcal mol^-1^) indicating that this process should not been observed under reaction conditions, despite the fact that it is highly exothermic. The third process – E2 elimination leading finally to the CH_3_S-CH_2_-S^-^ anion (green line) – also requires relatively high energy (17.5 kcal mol^-1^). The primary S_N_@S reaction ionic product – CH_3_S^-^ anion – is significantly more basic than methylthio-nitromethane, so the secondary proton transfer takes place yielding finally CH_3_S-CH^(-)^-NO_2_ anion (*m/z* 106).

### Reactions of Difluoromethyl Anion

**Figure 4:**
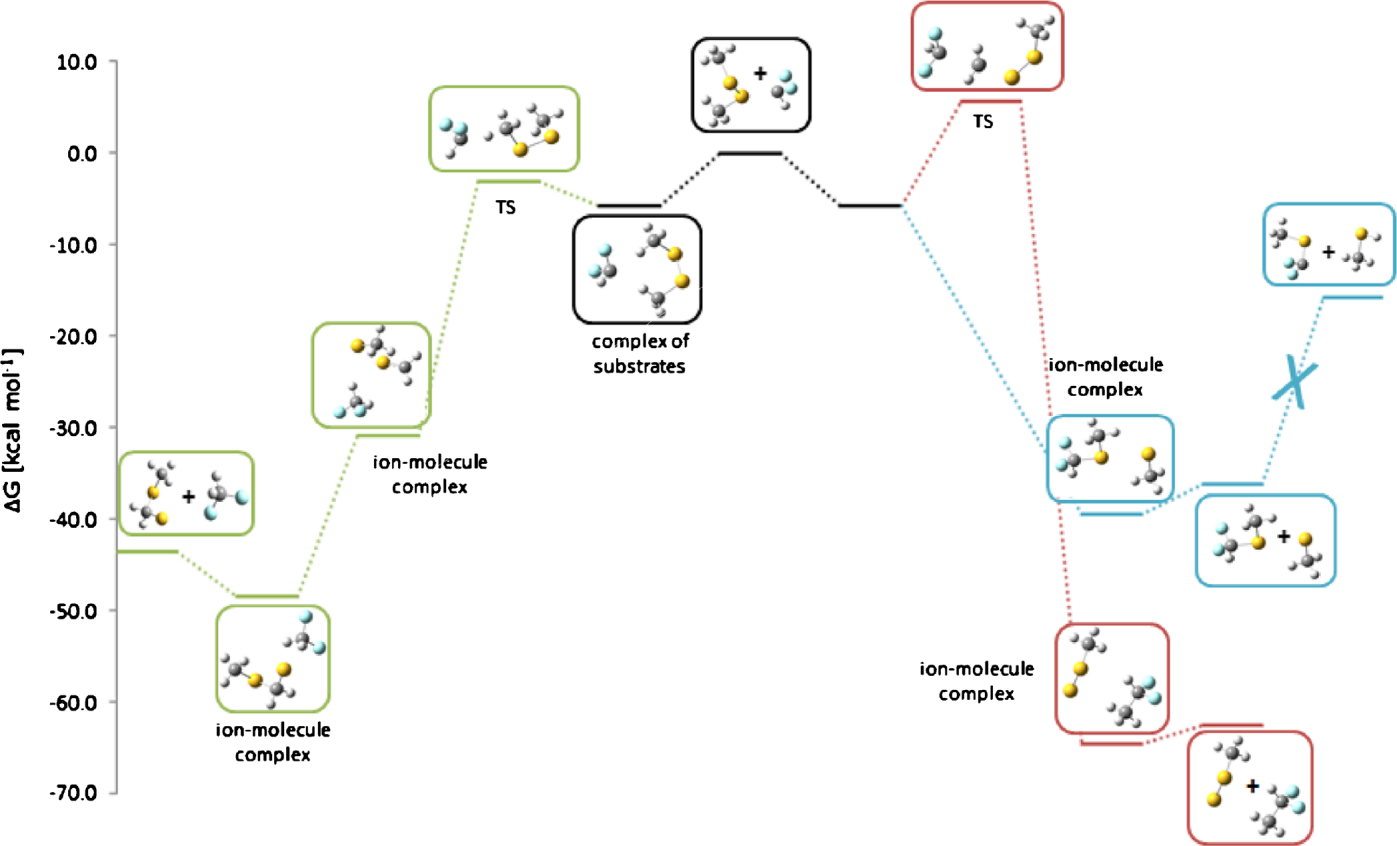
Calculated Gibbs free energy diagram of possible paths of the reaction between difluoromethyl anion and dimethyl disulfide in the gas phase. Blue trace: thiophilic reaction, red trace: S_N_2 reaction, green trace: elimination – addition reaction

On the other end of the basicity scale lies difluoromethyl anion (PA = 389 kcal mol^-1^). The spectrum recorded during its reaction with dimethyl disulfide shows that besides the expected thiophilic reaction (the presence of *m/z* 47 CH_3_S^-^ ion), also the formation of *m/z* 93 ion is observed. According to the proposal of Grabowski and Zhang [1], supported later by the calculations performed by Bachrach et al. [6], this ion should possess the hemithioacetal structure CH_3_S-CH_2_-S^-^. Our calculations (Figure 4) fully confirmed this structure. The reaction, which finally yields this ion, starts with the formation of the ion-molecule complex between difluoromethyl anion and dimethyl disulfide. Within this complex E2 elimination reaction takes place yielding the complex of difluoromethane, thioformaldehyde, and thiomethyl anion. Calculated geometry of this transition state shows the antiperiplanar attack of the CHF_2_^-^ anion respectively to the SCH_3_ fragment, which is typical for the E2 elimination mechanism. In the last step CH_3_S^-^ anion adds to the carbon atom of thioformaldehyde yielding final hemithioacetal anion. It is possible, of course, that the competitive dissociation of the ion-molecule complex takes place yielding thiomethyl anion.

The second reaction that has been observed is the S_N_@S process. It proceeds without any activation barrier. In a highly exoergic reaction the ion-molecule complex of difluoromethyl methyl sulfide and thiomethyl anion is formed, which eventually decomposes into free components. Because the proton affinity of difluoro(methylthio)methyl anion is much higher than the PA of CH_3_S^-^ ion, no proton transfer is observed in this case and the thiomethyl anion is the final ionic product.

Theoretically, the S_N_2 reaction, which should yield CH_3_-S-S^-^ anion (*m/z* 79) is possible but such ion has not been observed in the spectrum. Calculations show that in contrast to two abovementioned processes, S_N_2 reaction has an activation barrier of about 6 kcal mol^-1^ so it should be much slower.

### Reactions of Acetylenic Carbanions

**Scheme 5:**
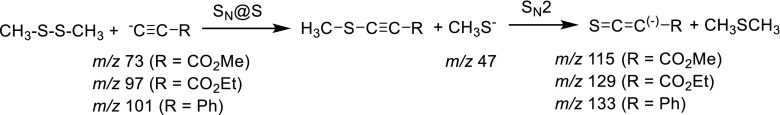
Reactions of substituted acetylenide anions with Me_2_S_2_ in the gas phase

In the next set of experiments, the reactions of acetylenic carbanions ^-^C≡C-R (R = CO_2_Me, CO_2_Et, and Ph) with dimethyl disulfide have been studied. In all cases only one two-step reaction path has been observed (Scheme 5). In the first step typical S_N_@S reaction took place yielding thiomethyl anion and the neutral product of the thiomethylation of the starting carbanion. The latter can undergo S_N_2 reaction with MeS^-^ yielding the respective thioketene anion and dimethyl sulfide.

In the case of the anion of methyl acetylenecarboxylate, another secondary S_N_2 reaction yielding the product of the same mass is possible, i.e., the attack of the thiomethyl anion on the methyl group of the ester moiety:

$$ {\mathrm{H}}_3\mathrm{C}-\mathrm{S}-\mathrm{C}\equiv \mathrm{C}-{\mathrm{CO}}_2{\mathrm{CH}}_3+{\mathrm{CH}}_3{\mathrm{S}}^{-}\kern0.5em \to {\mathrm{H}}_3\mathrm{C}-\mathrm{S}-\mathrm{C}\equiv \mathrm{C}-{{\mathrm{CO}}_2}^{-}+{\mathrm{CH}}_3{\mathrm{S}\mathrm{CH}}_3 $$

**Figure 5:**
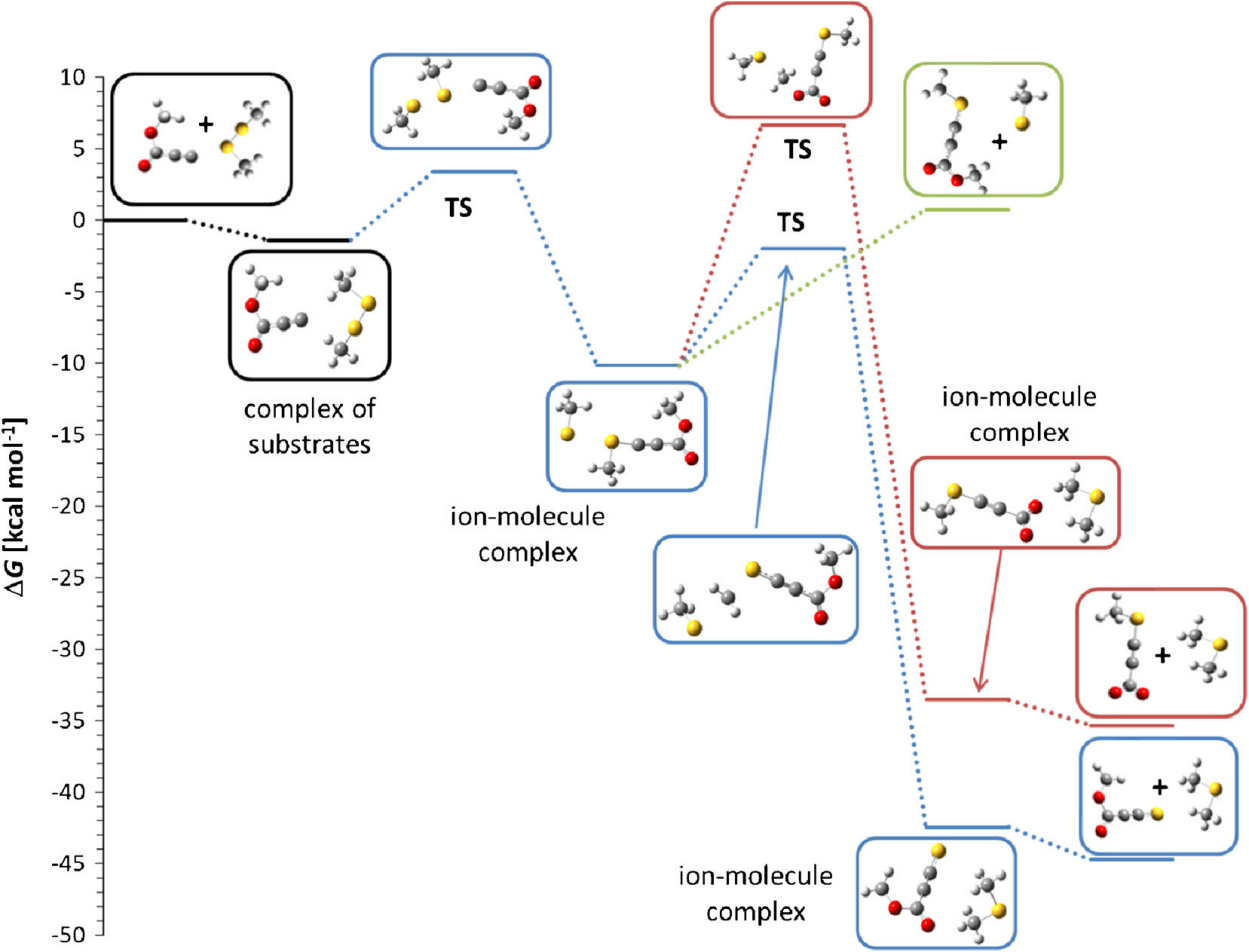
Calculated Gibbs free energy diagram of the thiophilic path of the reaction between methyl acetylenecarboxylate anion and dimethyl disulfide in the gas phase. Blue and green traces: thiophilic reaction, red trace: S_N_2 reaction

To solve this problem, the reaction with ethyl acetylenecarboxylate anion has been performed. In this case no attack on the ethyl group has been observed indicating clearly that the secondary S_N_2 reaction takes place solely on the thiomethyl group. The same conclusions come from the modeling of the reaction profiles (Figure 5). For clarity, calculated profiles of E2 and S_N_2 reactions are not shown. These processes have high activation free energy (16.3 and 21.8 kcal mol^-1^, respectively) and their products are not observed in the spectrum.

The thiophilic reaction starts with the formation of the complex of substrates, which, after passing a low activation barrier (3.4 kcal mol^-1^), is transformed into an ion-molecule complex of the thiomethylated methyl acetylenecarboxylate and thiomethyl anion (Δ*G* = –10.2 kcal mol^-1^). This complex can either dissociate yielding free thiomethyl anion as the ionic product (green trace in Figure 5) or can undergo S_N_2 reaction at the thiomethyl group (Δ*G*^≠^ = 8.2 kcal mol^-1^ relative to the complex) yielding substituted thioketene anion and dimethyl sulfide. This reaction is highly exoergic. An alternative S_N_2 reaction at the ester methyl group has much higher activation free energy (16.8 kcal mol^-1^ relative to the complex) so it is several orders of magnitude slower than the reaction at the thiomethyl group.

### Reactions of (Ethoxycarbonyl)methyl Anion, Ketene anion, and Carboxymethyl Anion

Standard ESI spectrum of monoethyl malonate anion (*m/z* 131) shows that there are three carbanions generated in the electrospray ion source: (ethoxycarbonyl)methyl anion (*m/z* 87), ketene anion (*m/z* 41), and carboxymethyl anion (*m/z* 59).

The main peak in the spectrum of the reaction products comes from the CH_3_S^-^ ion (*m/z* 47), indicating that the thiophilic reaction is the main process. The abundant presence of the *m/z* 133 peak shows that also the secondary proton exchange process takes place yielding the anion of ethyl (methylthio)acetate. The yield of this reaction is high because the proton affinity of the methylthio anion is slightly higher than that of the anion of ethyl (methylthio)acetate (PA = 353.5 kcal mol^-1^). Finally, traces of CH_3_SS^-^ ion (*m/z* 79) are observed.

However, in the product ion mass spectrum traces of ketene anion and carboxymethyl anion can be observed, suggesting that some of the product ions (namely CH_3_S^-^ and CH_3_SS^-^) may be the products of their reaction with dimethyl disulfide. To solve this problem, not only theoretical calculations were performed but also reactions of both additional carbanions with dimethyl disulfide were conducted.

**Figure 6:**
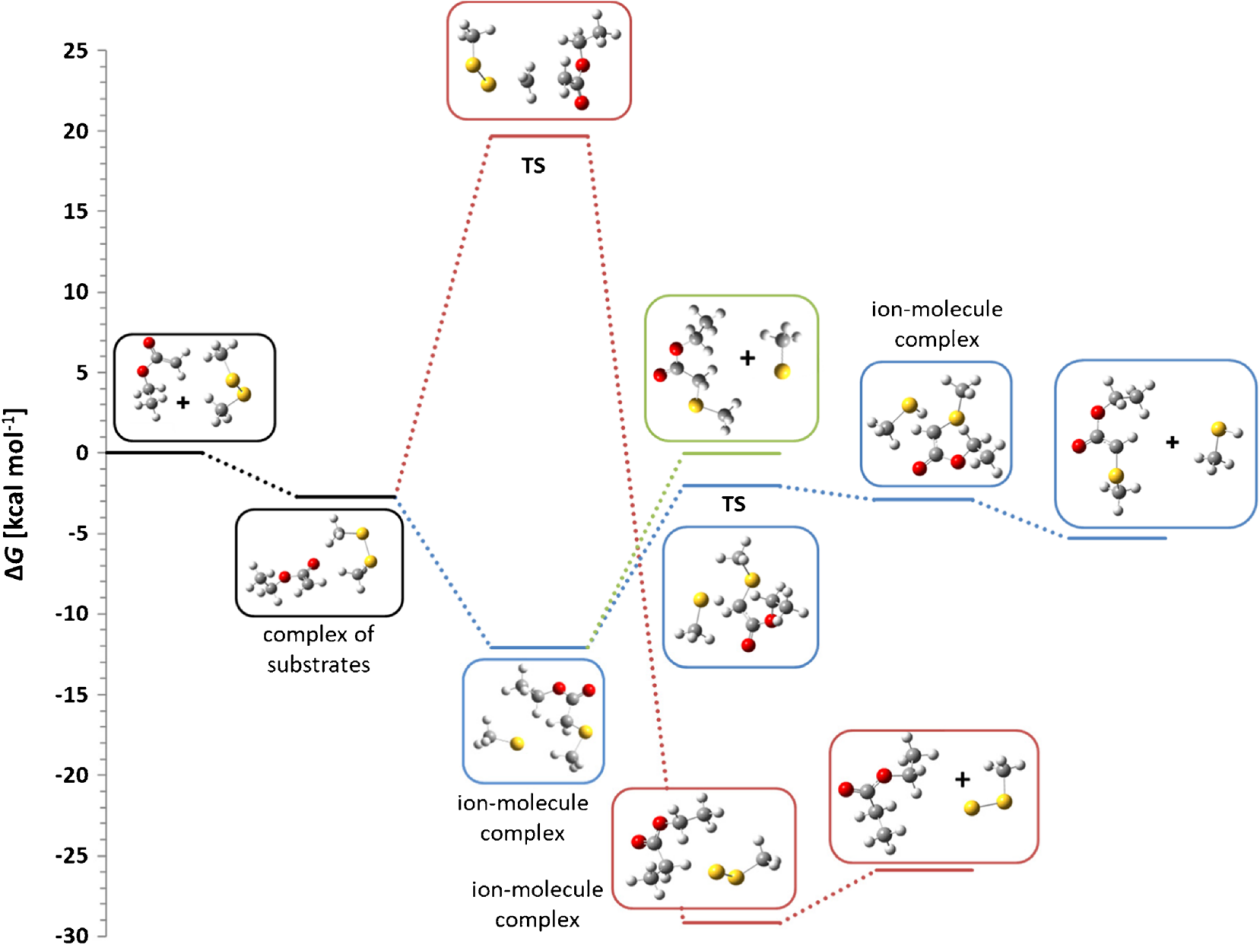
Calculated Gibbs free energy diagram of possible paths of the reactions between (ethoxycarbonyl)methyl anion and dimethyl disulfide in the gas phase. Blue and green traces: thiophilic reaction, red trace: S_N_2 reaction

Calculated Gibbs free energy profiles (Figure 6; the profile of the elimination – addition reaction, which products are not observed in the spectrum, is omitted) are in agreement with experimental results, suggesting that all observed product ions may be generated in reaction between (ethoxycarbonyl)methyl anion and Me_2_S_2_. In the first, exoergic step of thiophilic reaction (blue line), ion-molecule complex is formed (Δ*G* = –10.2 kcal mol^-1^), which can either dissociate or subsequent proton transfer reaction, requiring low activation free energy, can occur. S_N_2 reaction (red line) has very high activation barrier (Δ*G*^≠^ = 19.9 kcal mol^-1^) so it should be much slower (which would explain very low abundance in the product ion mass spectrum) or not occur at all.

**Figure 7:**
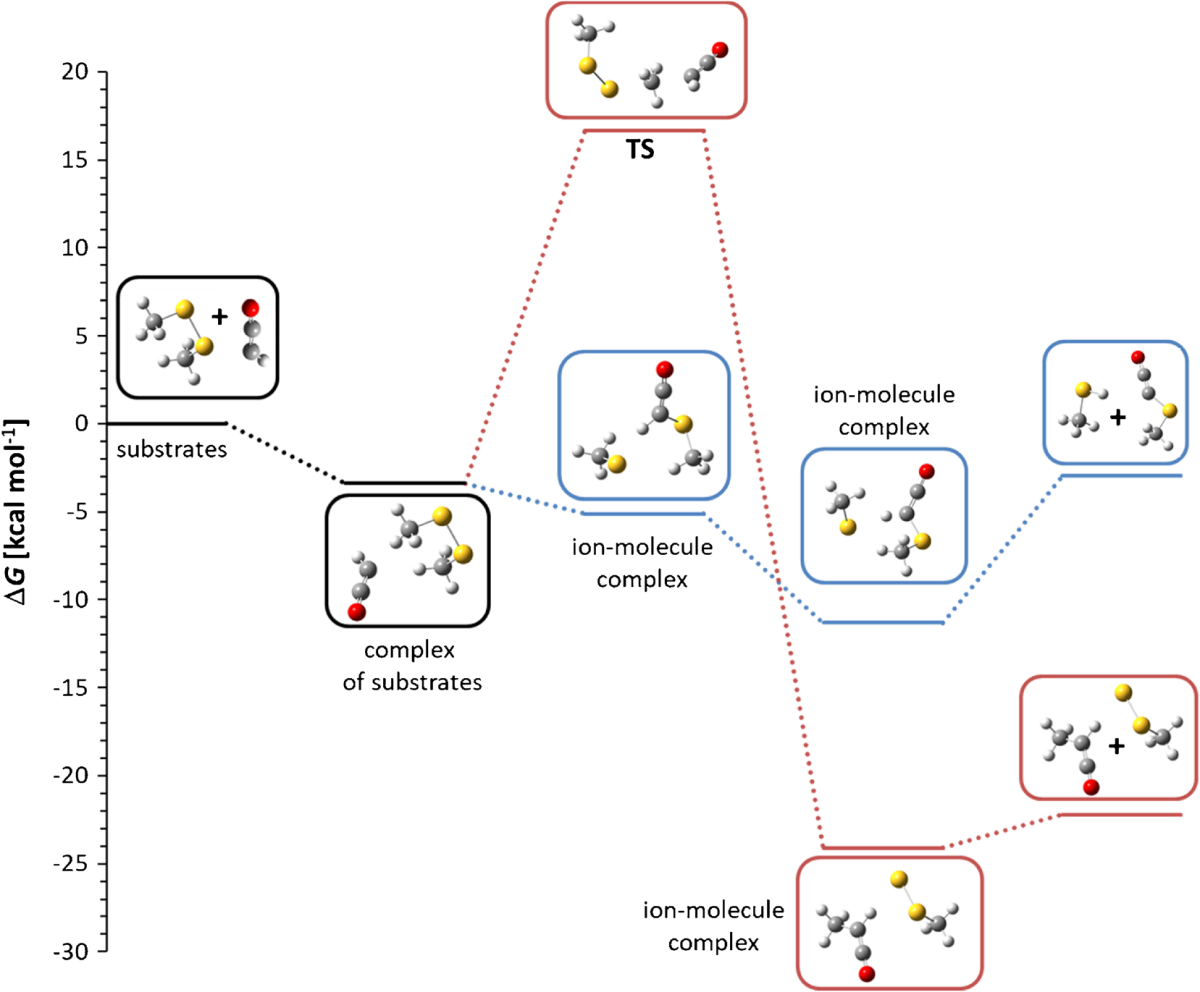
Calculated Gibbs free energy diagram of possible paths of the reaction between ketene anion and dimethyl disulfide in the gas phase. Blue trace: thiophilic reaction, red trace: S_N_2 reaction

In the next experiment the reaction of ketene anion with dimethyl disulfide has been studied. The main observed peak is CH_3_S^-^ (*m/z* 47) indicating that also for this carbanion thiophilic reaction is the main reaction pathway. This reaction does not stop at the step of the formation of methylthio anion and (methylthio)ethenone, but subsequent proton transfer reaction occurs (presence of *m/z* 87 ion: CH_3_S-C^(-)^=C=O). Finally, traces of CH_3_SS^-^ ion (*m/z* 79) can also be observed. The same conclusions come from the modeling of the reaction profiles (Figure 7). For thiophilic reaction, complex of substrates (Δ*G* = –3.4 kcal mol^-1^) is transformed into ion-molecule complex (Δ*G* = –5.1 kcal mol^-1^) without an activation barrier. This complex can either dissociate yielding free thiomethyl anion as the ionic product or can undergo proton transfer reaction yielding another ion-molecule complex of (methylthio)ethenone and methyl sulfide (Δ*G* = –2.3 kcal mol^-1^). S_N_2 reaction again is highly exoergic, but has high activation barrier (Δ*G*^≠^ = 16.7 kcal mol^-1^), explaining very low intensity of the product peak in mass spectrum.

**Figure 8:**
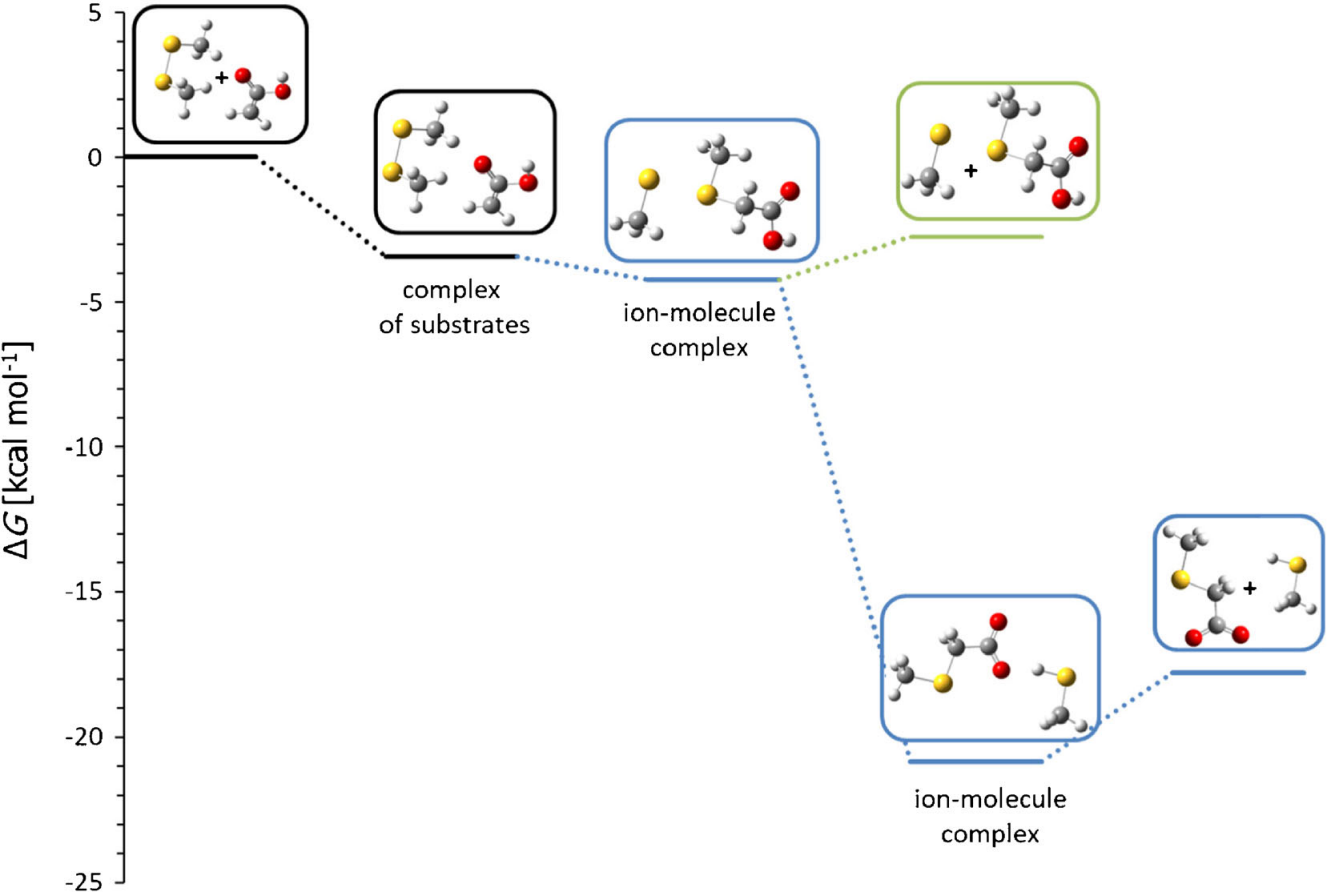
Calculated Gibbs free energy diagram of possible paths of the reaction between carboxymethyl anion and dimethyl disulfide in the gas phase

A comment should be made concerning the structure of *m/z* 87 ion. It can be described by two mesomeric formulas:





Our calculations showed that the geometry of this anion is described better by the MeS-C^(-)^=C=O mesomeric structure rather than MeS-C≡C-O^-^. The S-C-C angle is equal to 142° so it is much closer to 120° than 180° characteristic to the acetylenic structure.

Finally, reaction of carboxymethyl anion with dimethyl disulfide was conducted. The main peak in the spectrum of the reaction products is the *m/z* 105 ion (CH_3_S-CH_2_CO_2_^-^), indicating that the thiophilic reaction with subsequent proton transfer is the main process taking place in a collision chamber. Peak of methylthio anion (*m/z* 47) has very low intensity, which suggests that subsequent reaction is very favorable. No other peaks were observed. Calculated Gibbs free energy confirms experimental results.

Taking into consideration experimental and theoretical results obtained for ketene anion and carboxymethyl anion, we argue that product ion peaks present in (ethoxycarbonyl)methyl anion spectrum are products of the reaction of the latter anion with dimethyl disulfide.

### Single Electron Transfer (SET) Mechanism of the Thiophilic Reaction – is it Possible?

The last point, which has to be discussed concerning the studied reactions, is the possibility of an alternative thiophilic reaction mechanism involving single electron transfer (SET) in the first step. In principle, it is possible that the carbanion will deliver one electron to the neutral substrate forming a radical–radical anion pair, which can react further giving the same products as during direct S_N_@S reaction. Reactions involving SET are quite common in organic chemistry [29] so it was interesting to see whether such mechanism is possible for the reactions of carbanions with dimethyl disulfide. The simplest way to solve this problem is to calculate electron affinity (EA) of dimethyl disulfide and radicals relative to the carbanions used in this work. A few test calculations performed for radicals with known experimental EA values showed that it was necessary to use G4(MP2) composite method because DFT methods as well as MP2 ab initio method, even with large basis sets, did not give acceptable results. Calculated EA values are given in Table 2. They show that the electron transfer from all studied carbanions to dimethyl disulfide molecule is endothermic. Therefore this reaction channel is very unlikely, taking into account that for the majority of the studied carbanions the thiophilic reaction proceeds without an activation barrier and for a few carbanions with lowest proton affinity this barrier is marginal.

**Table 2:** Experimental [28] and Calculated Using G4(MP2)Method Electron Affinities (in eV) of Selected Radicals and Dimethyl Disulfide

	EA G4(MP2)	EA exp.
^●^CH_2_NO_2_	2.48	2.48
^●^CCl_3_	2.05	2.16
^●^CH_2_COCH_3_	1.75	1.75
^●^CH_2_COOMe	1.61	1.80
^●^CH_2_CN	1.58	1.53
^●^CHF_2_	0.72	1.21
[CH_3_SSCH_3_]^- ●^	0.16	-

## Conclusions

Mass spectrometry experiments together with DFT calculations showed that dimethyl disulfide can react with aliphatic carbanions in the gas phase according to three mechanisms, depending on the structure of the carbanion and its proton affinity. The first reaction path, observed for all studied carbanions, is the nucleophilic substitution at the sulfur atom called the thiophilic reaction (S_N_@S). It results in the formation of the thiomethyl anion CH_3_S^-^ (*m/z* 47). The neutral product of this reaction – the thiomethylated carbanion – can in some cases react further with the thiomethyl anion yielding the secondary ionic products. In the case of the carbanions possessing strong electron-withdrawing groups, the resulting product is acidic enough to be deprotonated by CH_3_S^-^ anion. On the other hand, thiomethylated anions of acetylenecarboxylic acid esters undergo S_N_2 reaction with CH_3_S^-^ yielding dimethyl sulfide and the anion of substituted thioketene. Calculated transition states for this reaction pathway are located close to the energy level of complex of substrates, indicating that this reaction proceeds without a significant activation barrier. Modeled Gibbs free energy profiles suggest that thiophilic reaction is an energetically favorable process.

The second reaction is the S_N_2 nucleophilic substitution, in which the methyl disulfide anion (CH_3_SS^-^, *m/z* 79) is the leaving group. This reaction, despite its high exothermicity, is characterized according to the results of our calculations by high activation energy, so it is observed as a minor process in the case of only four studied carbanions.

The formation of the third observed ionic product with *m/z* 93 can be rationalized, as described earlier by Grabowski and Zhang, by the E2 elimination resulting in the formation of CH_3_S^-^ anion and thioformaldehyde molecule followed by addition of the CH_3_S^-^ to CH_2_=S yielding CH_3_S-CH_2_-S^-^ anion. This process has been observed only for three carbanions with the PA values at least 375 kcal mol^-1^. Calculated geometry of the transition states of these reactions shows the antiperiplanar attack of the nucleophile anion respectively to the SCH_3_ fragment, which is typical for the E2 elimination mechanism.

Our results confirmed that mass spectrometry together with quantum chemical calculations is a powerful tool for studying gas-phase reactions between anions and electrophilic species.

## Electronic supplementary material

ESM 1 (DOCX 2054 kb)
