# Supplementary material for: Gas-Phase Reactions of Dimethyl Disulfide with Aliphatic Carbanions - A Mass Spectrometry and Computational Study
Source: J Am Soc Mass Spectrom. 2018 Jan 8;29(3):588–99. doi: 10.1007/s13361-017-1858-x (PMC5838211; doi:10.1007/s13361-017-1858-x)
Supplement: Supplementary file 1 — (DOCX 2054 kb) [file 13361_2017_1858_MOESM1_ESM.docx]

**Gas-Phase Reactions of Dimethyl Disulfide with Aliphatic Carbanions – A Mass Spectrometry and Computational Study**

**Supporting Information**

Barbara Franczuk and Witold Danikiewicz

Institute of Organic Chemistry, Polish Academy of Sciences, Kasprzaka 44/52, 01-224 Warsaw, Poland

| **Table of Contents** | |
| --- | --- |
|  |  |
| **Calculations at PBE1PBE/6-311+G(2d,p) level of theory:** |  |
| Calculated enthalpies for reactions between carbanions with Me_2_S_2_ in the gas phase | **S1** |
| Calculated free Gibbs energy for reactions between carbanions with Me_2_S_2_ in the gas phase | **S2** |
| Enthalpy (Δ*H*) profiles of the reactions between ^-^CH_2_NO_2_ and Me_2_S_2_ | **S3** |
| Enthalpy (Δ*H*) profiles of the reactions between ^-^CCl_3_ and Me_2_S_2_ | **S4** |
| Gibbs free energy (Δ*G*) profiles of the reactions between ^-^CCl_3_ and Me_2_S_2_ | **S5** |
| Enthalpy (Δ*H*) profiles of the reactions between ^-^C≡C-CO_2_Me and Me_2_S_2_ | **S6** |
| Enthalpy (Δ*H*) profiles of the reactions between ^-^CH=C=O and Me_2_S_2_ | **S7** |
| Gibbs free energy (Δ*G*) profiles of the reactions between ^-^CH_2_COCH_3_ and Me_2_S_2_ | **S8** |
| Enthalpy (Δ*H*) profiles of the reactions between ^-^CH_2_COCH_3_ and Me_2_S_2_ | **S9** |
| Enthalpy (Δ*H*) profiles of the reactions between ^-^CH_2_CO_2_Et and Me_2_S_2_ | **S10** |
| Gibbs free energy (Δ*G*) profiles of the reactions between ^-^C≡C-Ph and Me_2_S_2_ | **S11** |
| Enthalpy (Δ*H*) profiles of the reactions between ^-^C≡C-Ph and Me_2_S_2_ | **S12** |
| Gibbs free energy (Δ*G*) profiles of the reactions between ^-^CH_2_CO_2_Me and Me_2_S_2_ | **S13** |
| Enthalpy (Δ*H*) profiles of the reactions between ^-^CH_2_CO_2_Me and Me_2_S_2_ | **S14** |
| Enthalpy (Δ*H*) profiles of the reactions between ^-^CH_2_CN and Me_2_S_2_ | **S15** |
| Gibbs free energy (Δ*G*) profiles of the reactions between ^-^CHCl_2_ and Me_2_S_2_ | **S16** |
| Enthalpy (Δ*H*) profiles of the reactions between ^-^CHCl_2_ and Me_2_S_2_ | **S17** |
| Gibbs free energy (Δ*G*) profiles of the reactions between ^–^CF_3_ and Me_2_S_2_ | **S18** |
| Enthalpy (Δ*H*) profiles of the reactions between ^–^CF_3_ and Me_2_S_2_ | **S19** |
| Enthalpy (Δ*H*) profiles of the reactions between ^–^CHF_2_ and Me_2_S_2_ | **S20** |
|  |  |
| **Mass Spectra for reactions of Me_2_S_2_ with carbanions:** |  |
| Product ion spectrum recorded for the reaction of Me_2_S_2_ with ^–^CCl_3_ | **S21** |
| Product ion spectrum recorded for the reaction of Me_2_S_2_ with ^-^C≡C-CO_2_Et | **S22** |
| Product ion spectrum recorded for the reaction of Me_2_S_2_ with ^-^CH_2_COCH_3_ | **S23** |
| Product ion spectrum recorded for the reaction of Me_2_S_2_ with ^–^CHCl_2_ | **S24** |
| Product ion spectrum recorded for the reaction of Me_2_S_2_ with ^–^CF_3_ | **S25** |

Table S2.Calculated enthalpies for reactions between carbanions with Me_2_S_2_ in the gas phase at PBE1PBE/6-311+G(2d,p), (all values in kcal mol^-1^).
PA of CH_3_SCH_2_S^-^ = 347.8 kcal mol^-1^


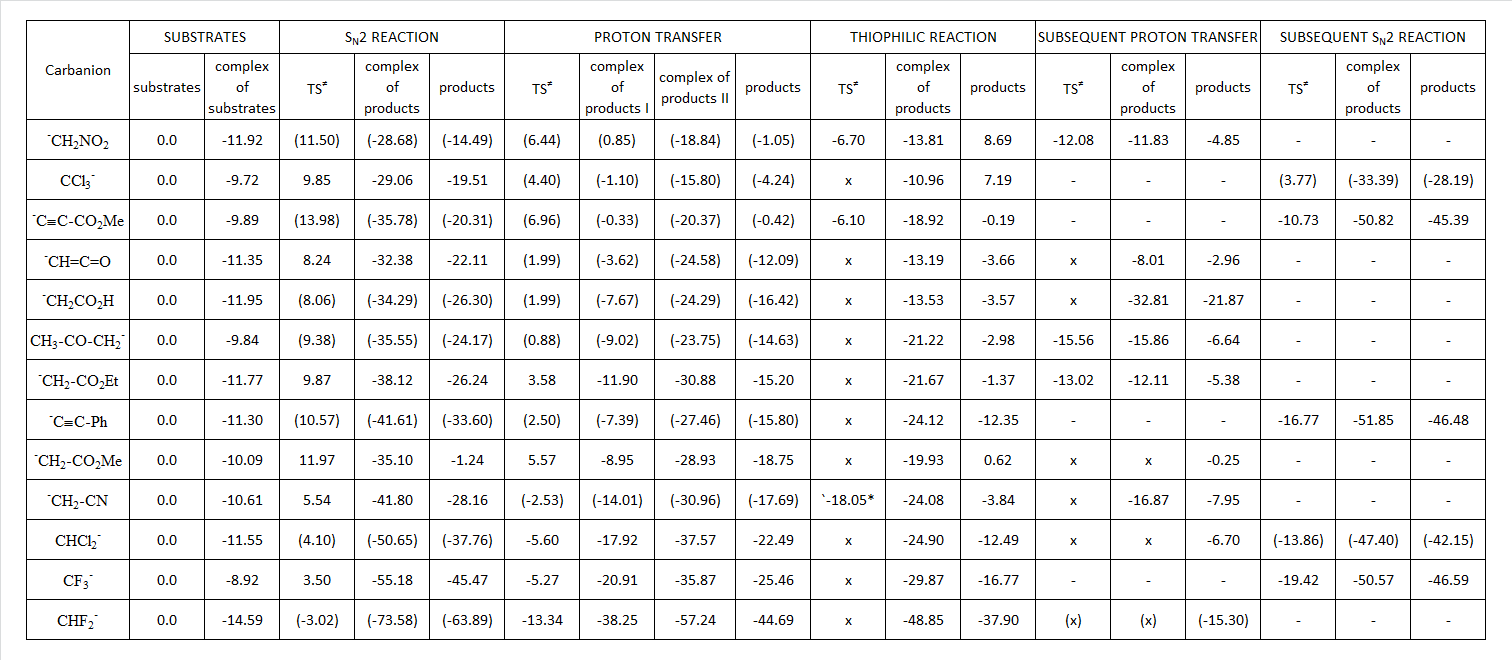


x - value not found

‘* - value for ion-molecule complex

values in the parenthesis - reaction does not occur in the gas phase

**Table S3.** Calculated free Gibbs energy for reactions between carbanions with Me_2_S_2_ in the gas phase at PBE1PBE/6-311+G(2d,p), (all values in kcal mol^-1^).


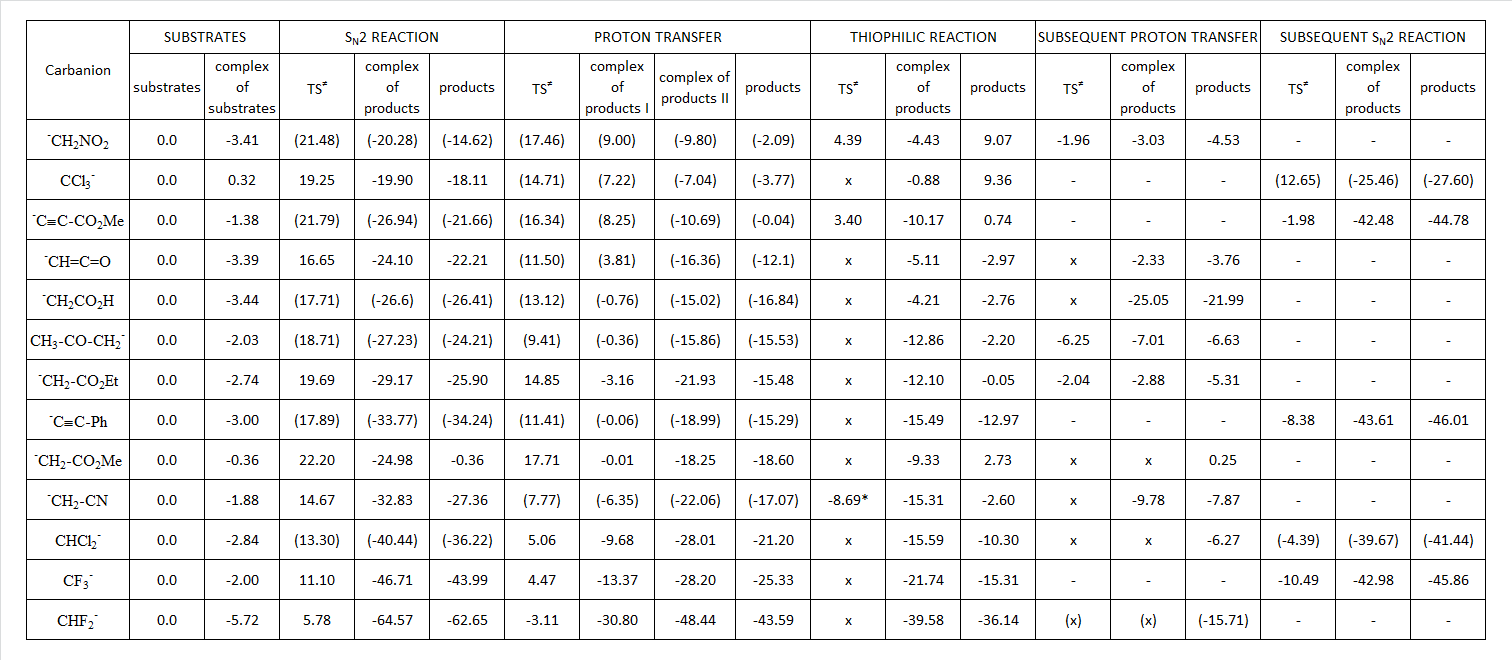


x - value not found

‘* - value for ion-molecule complex

values in the parenthesis - reaction does not occur in the gas phase


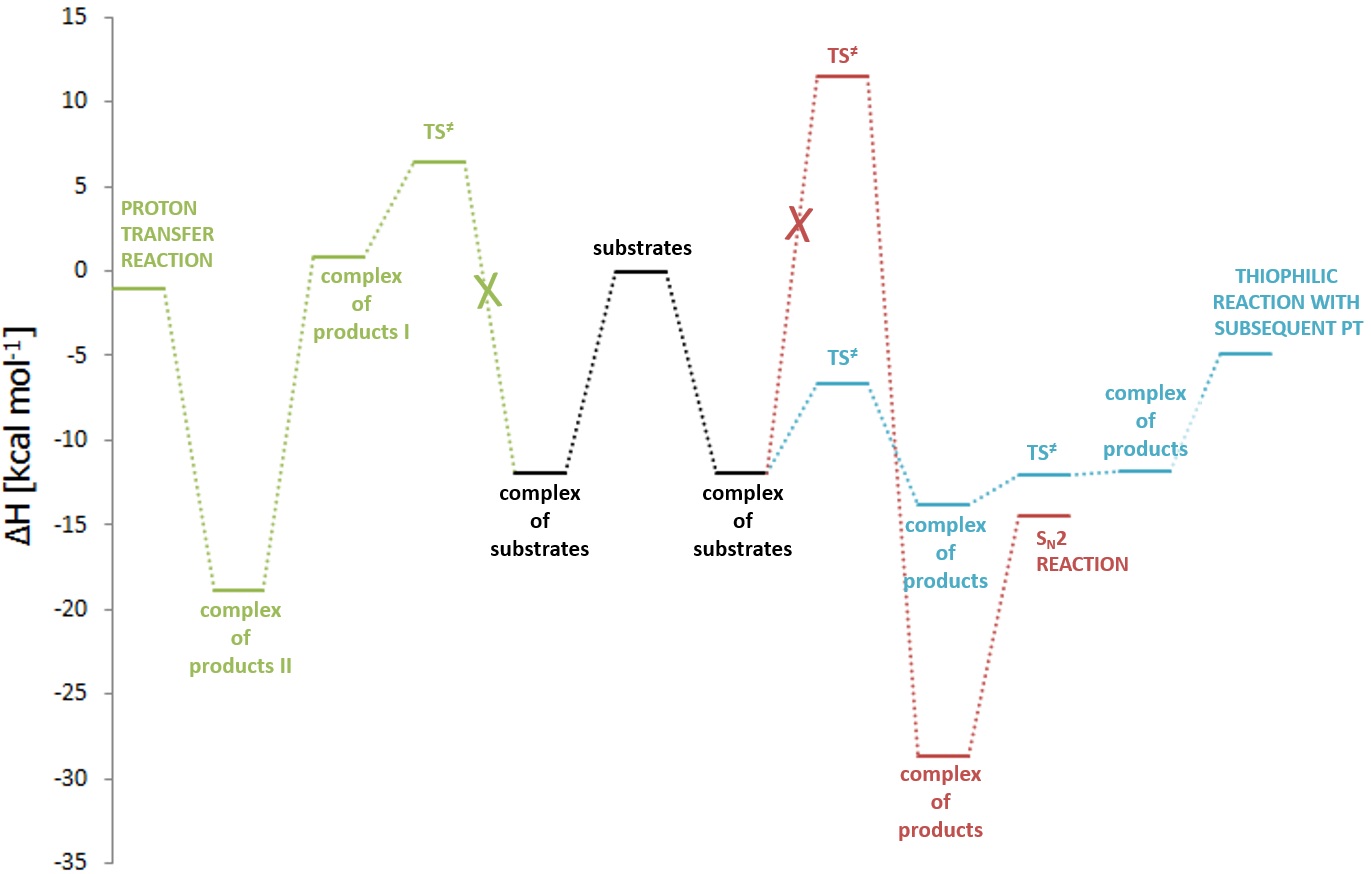


Figure S3. Enthalpy (Δ*H*) profiles of the reactions between ^-^CH_2_NO_2_ and Me_2_S_2_.


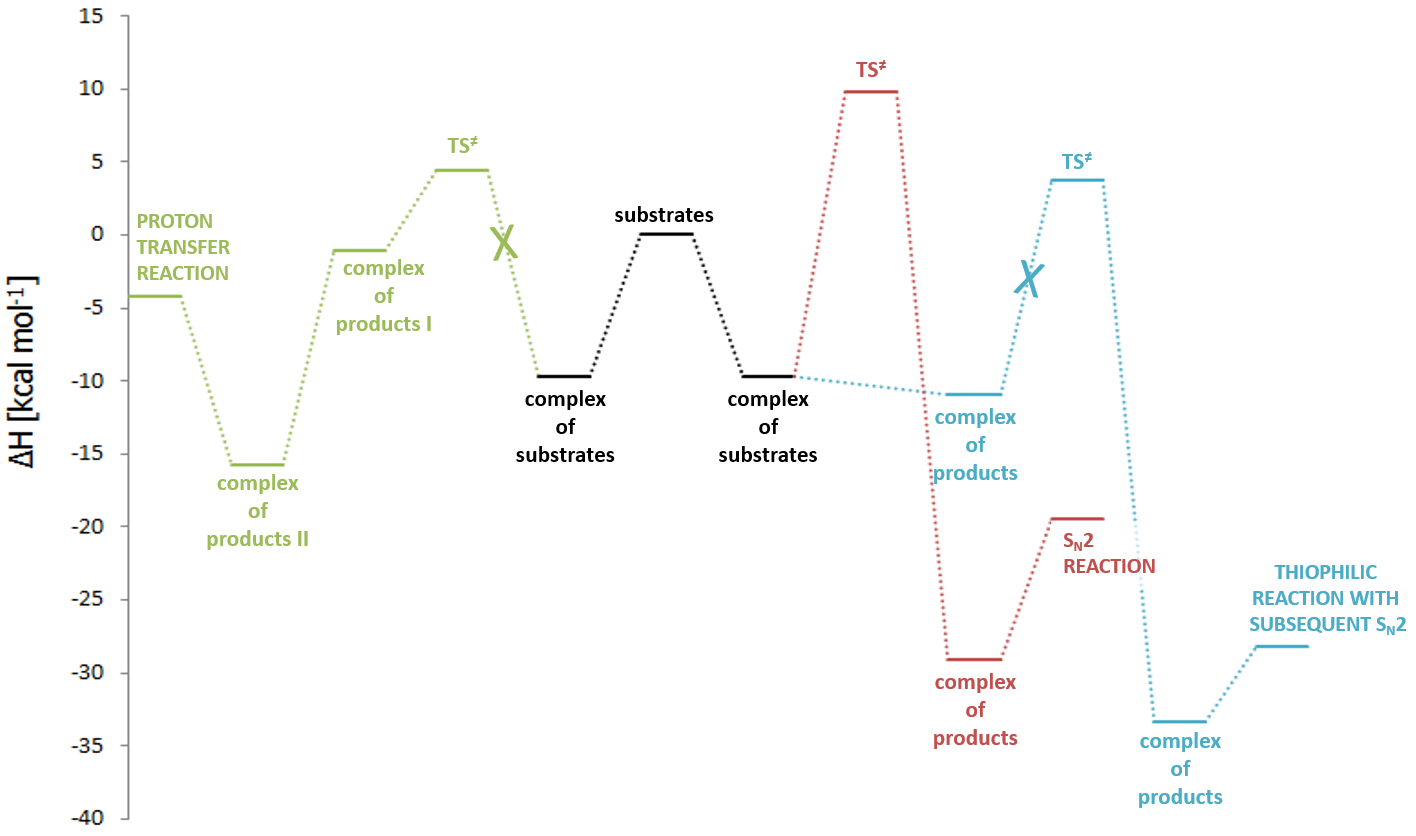


Figure S4. Enthalpy (Δ*H*) profiles of the reactions between ^-^CCl_3_ and Me_2_S_2_.


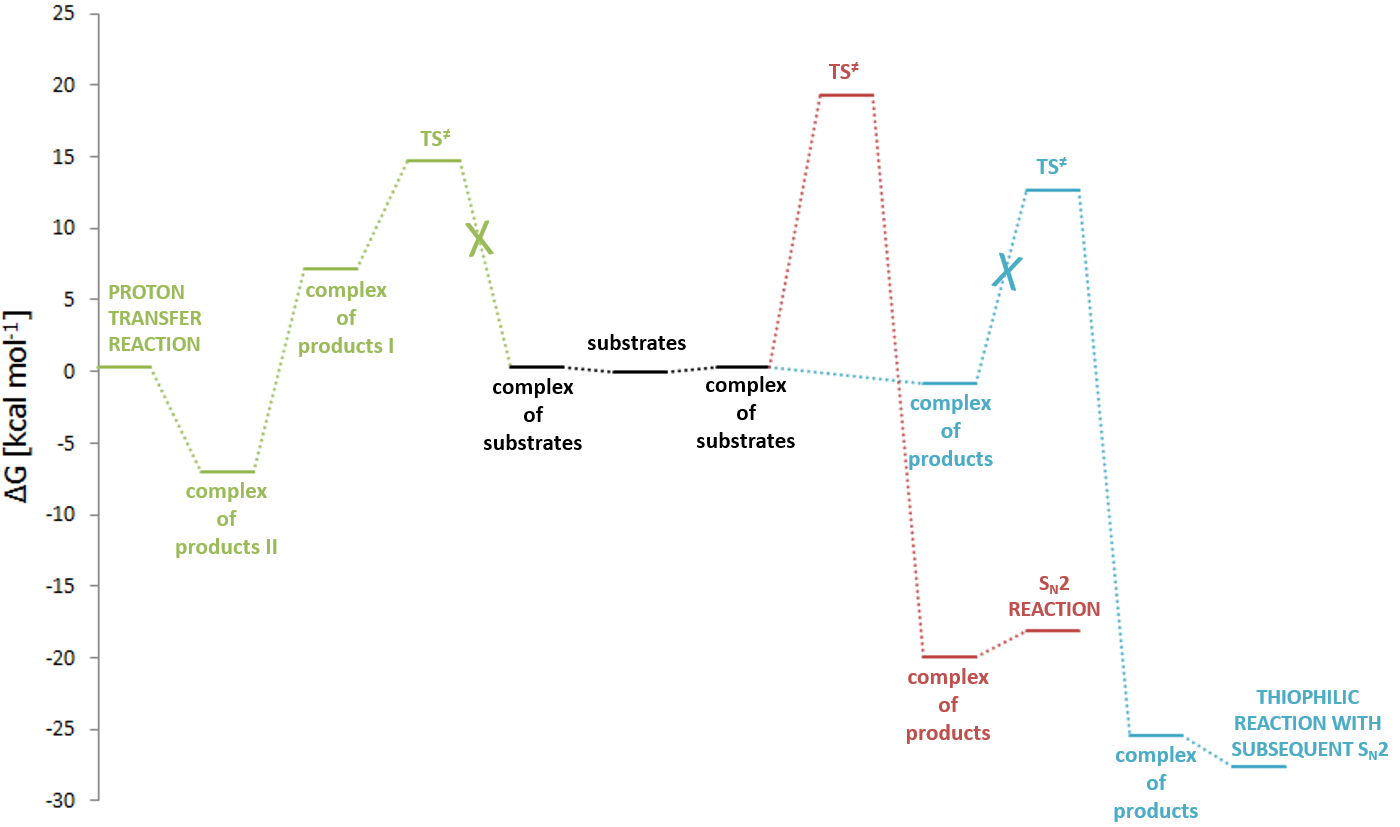


Figure S5. Gibbs free energy (Δ*G*) profiles of the reactions between ^-^CCl_3_ and Me_2_S_2_.


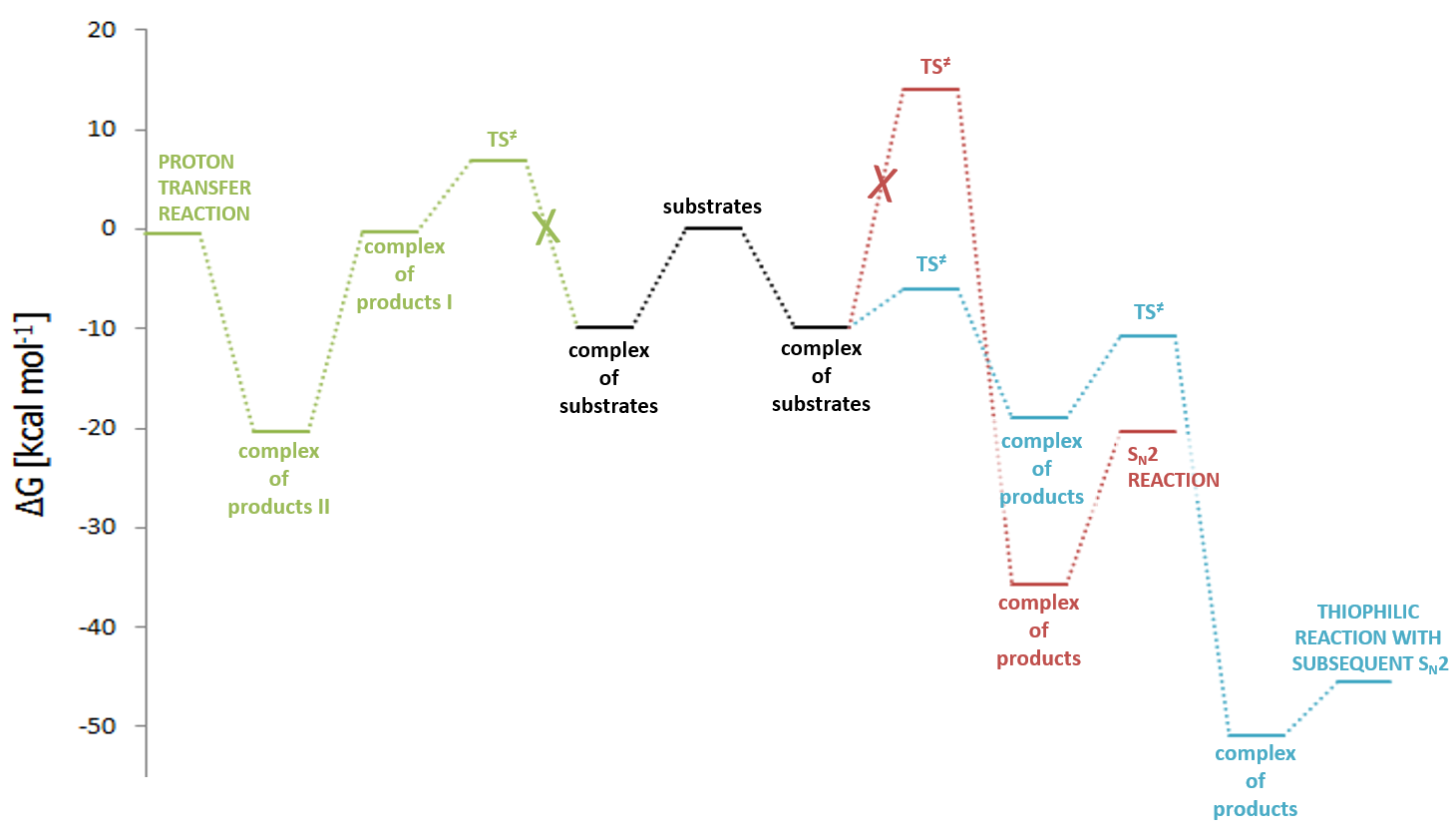


Figure S6. Enthalpy (Δ*H*) profiles of the reactions between ^-^C≡C-CO_2_Me and Me_2_S_2_.


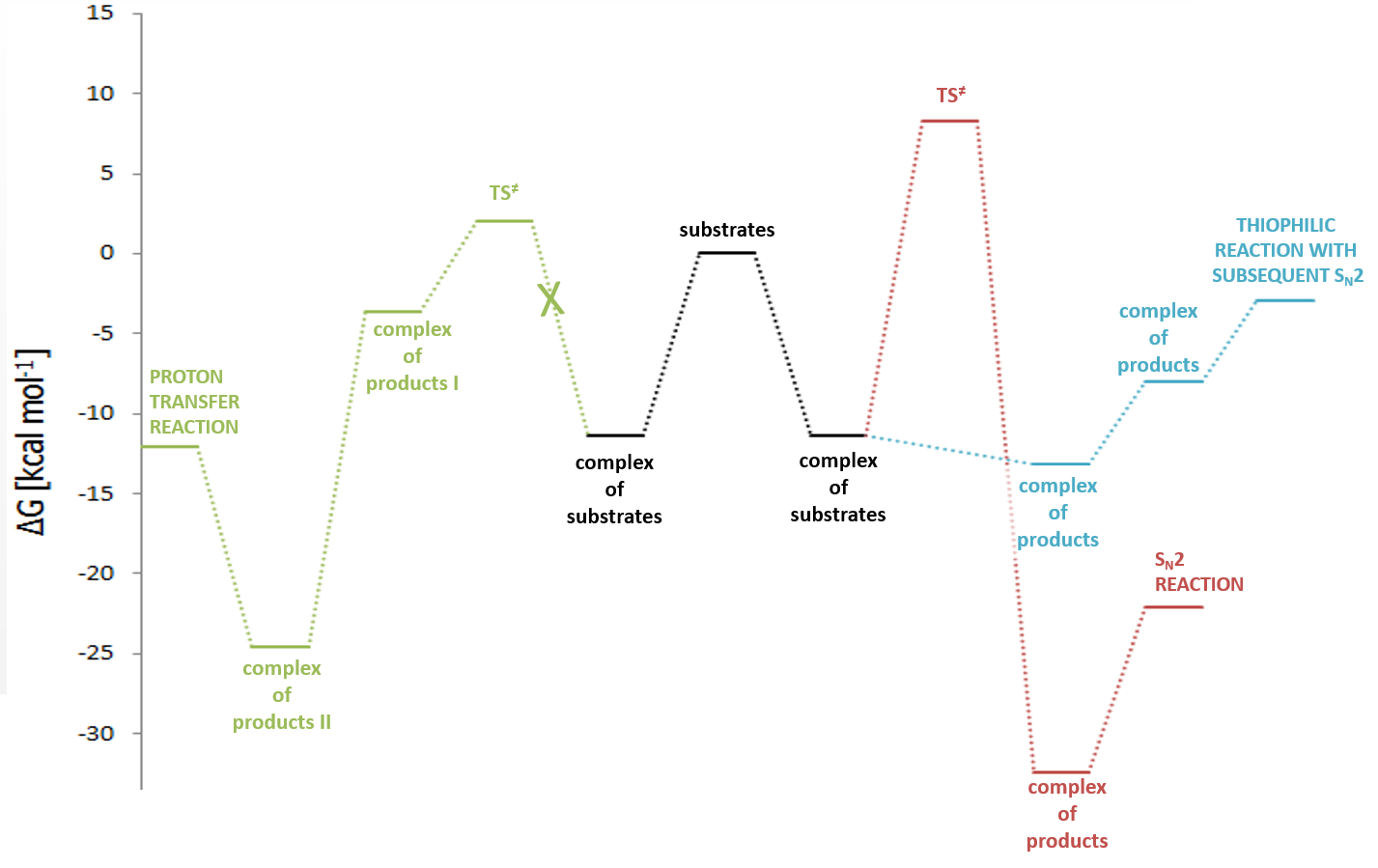


Figure S7. Enthalpy (Δ*H*) profiles of the reactions between ^-^CH=C=O and Me_2_S_2_


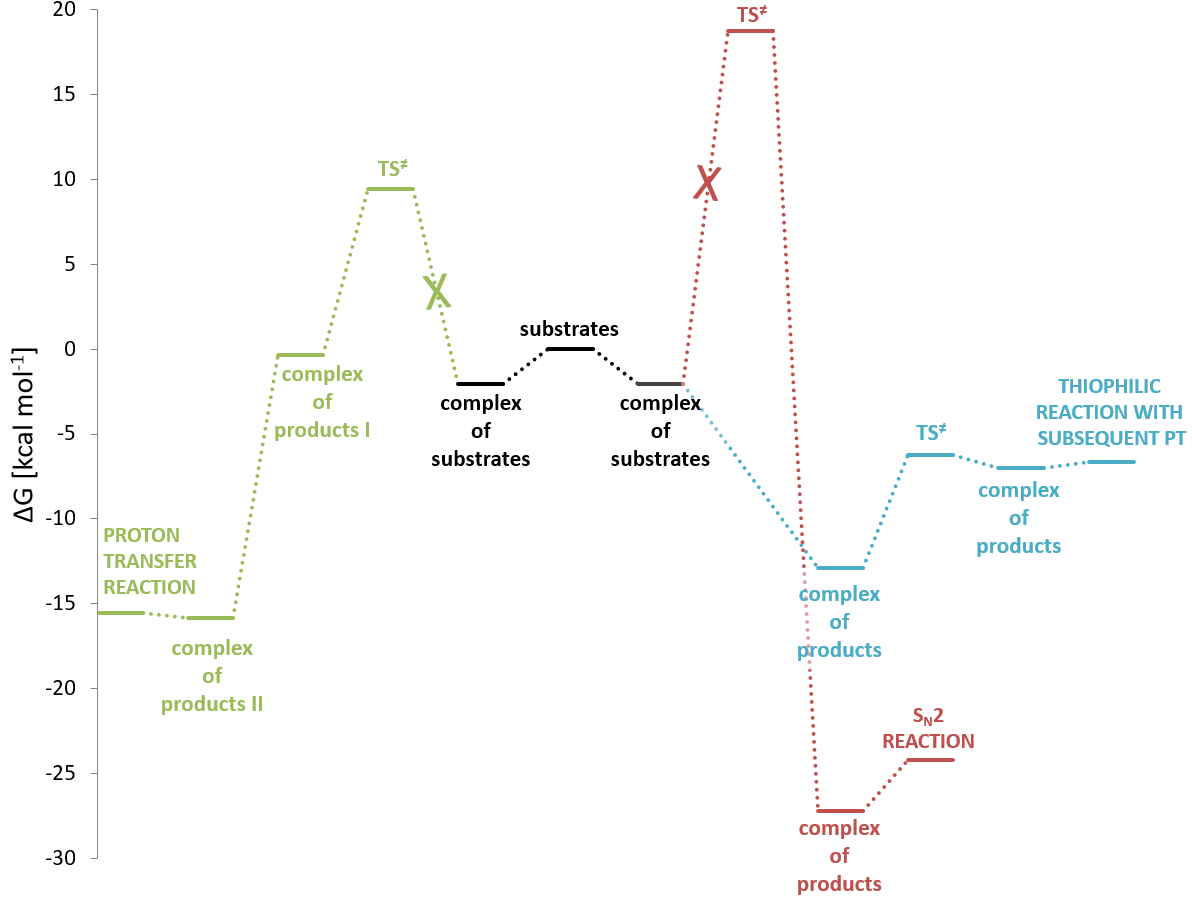


Figure S8. Gibbs free energy (Δ*G*) profiles of the reactions between ^-^CH_2_COCH_3_ and Me_2_S_2_


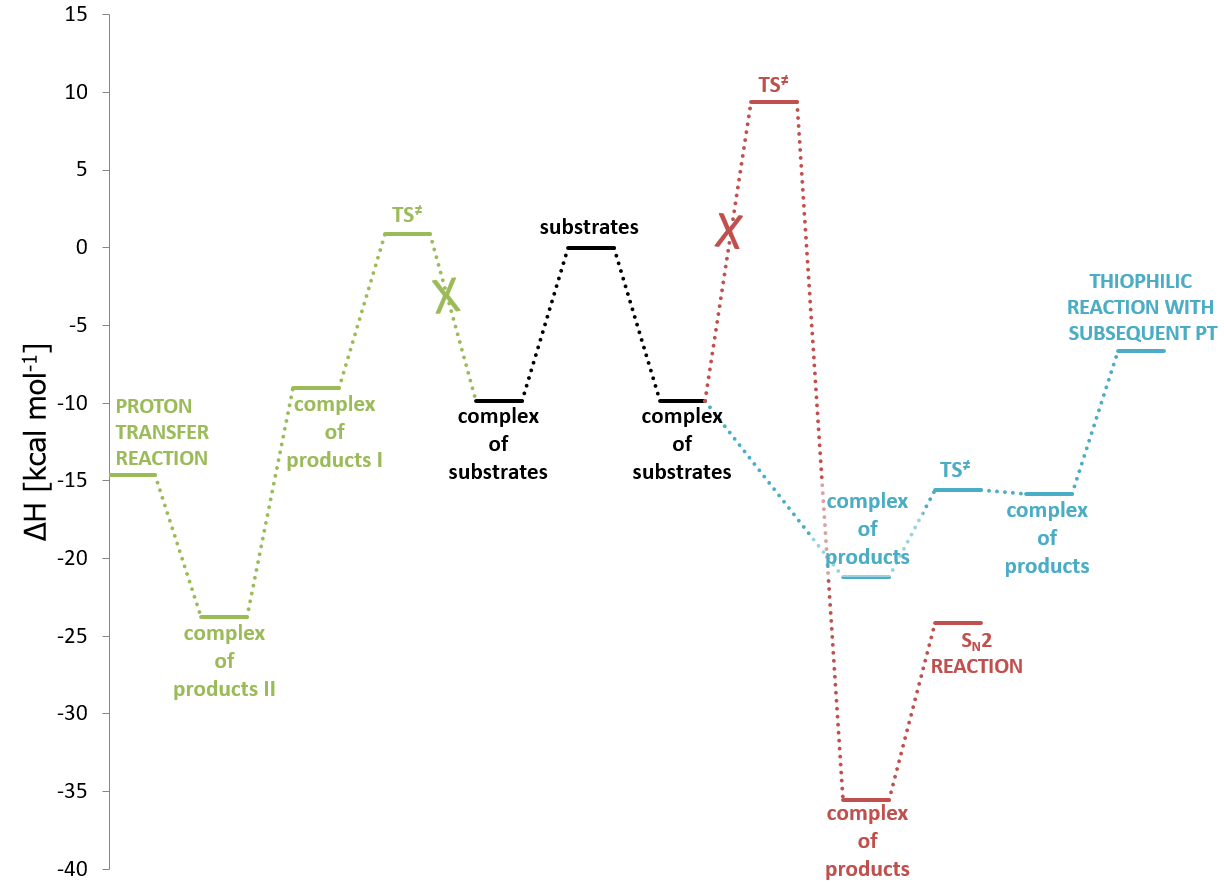


Figure S9. Enthalpy (Δ*H*) profiles of the reactions between ^-^CH_2_COCH_3_ and Me_2_S_2_


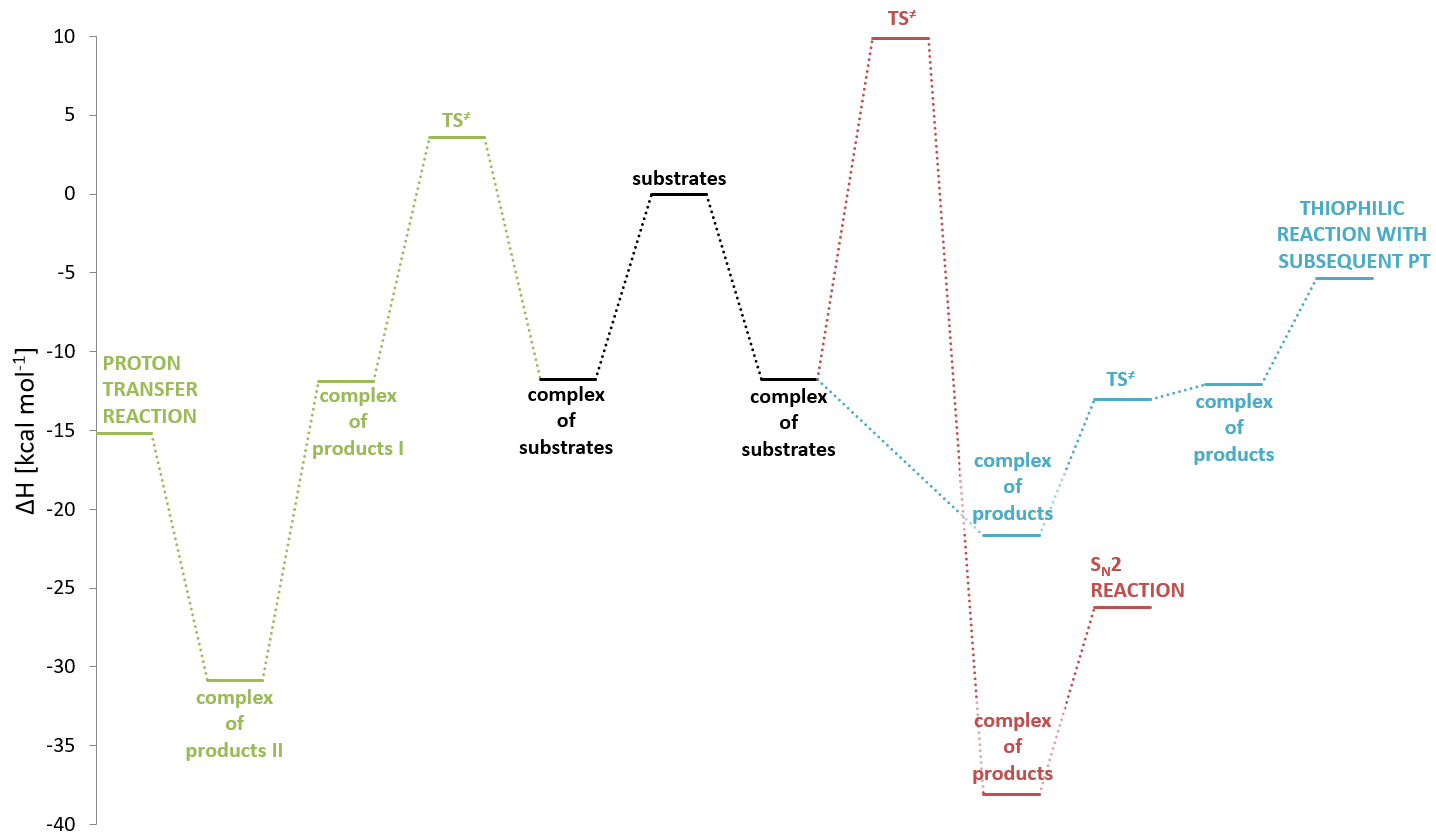


Figure S10. Enthalpy (Δ*H*) profiles of the reactions between ^-^CH_2_CO_2_Et and Me_2_S_2_


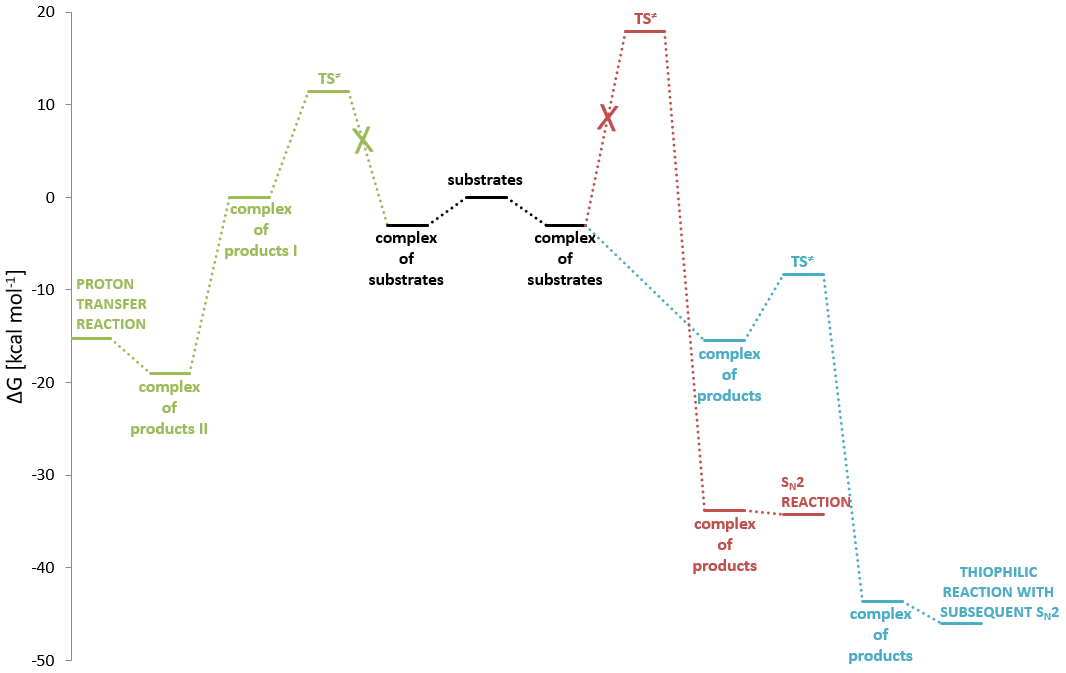


Figure S11. Gibbs free energy (Δ*G*) profiles of the reactions between ^-^C≡C-Ph and Me_2_S_2_


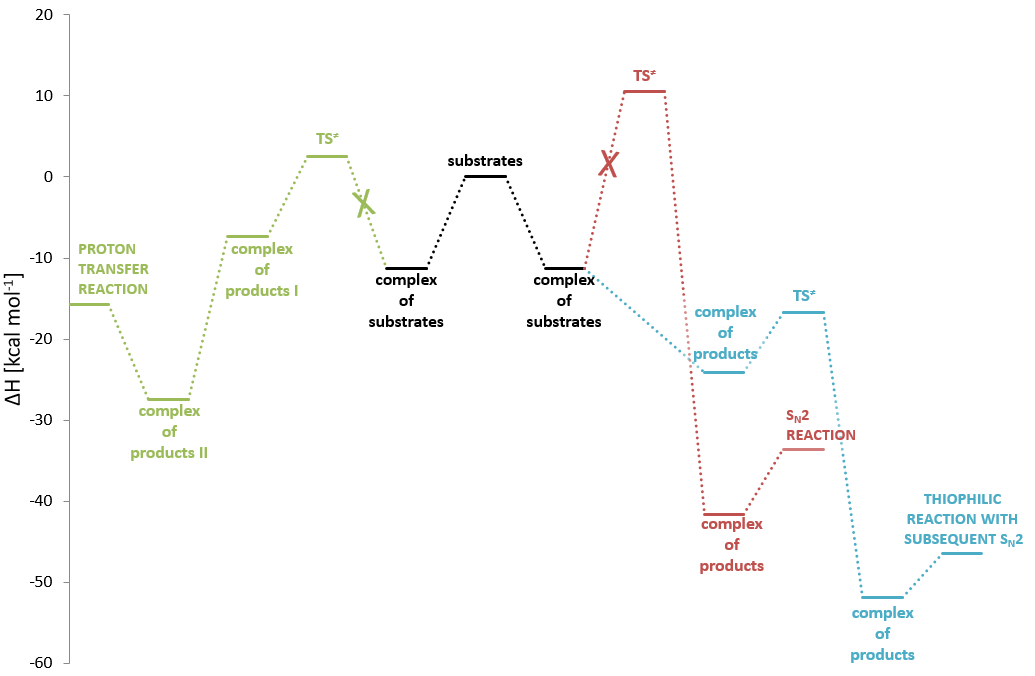


Figure S12. Enthalpy (Δ*H*) profiles of the reactions between ^-^C≡C-Ph and Me_2_S_2_


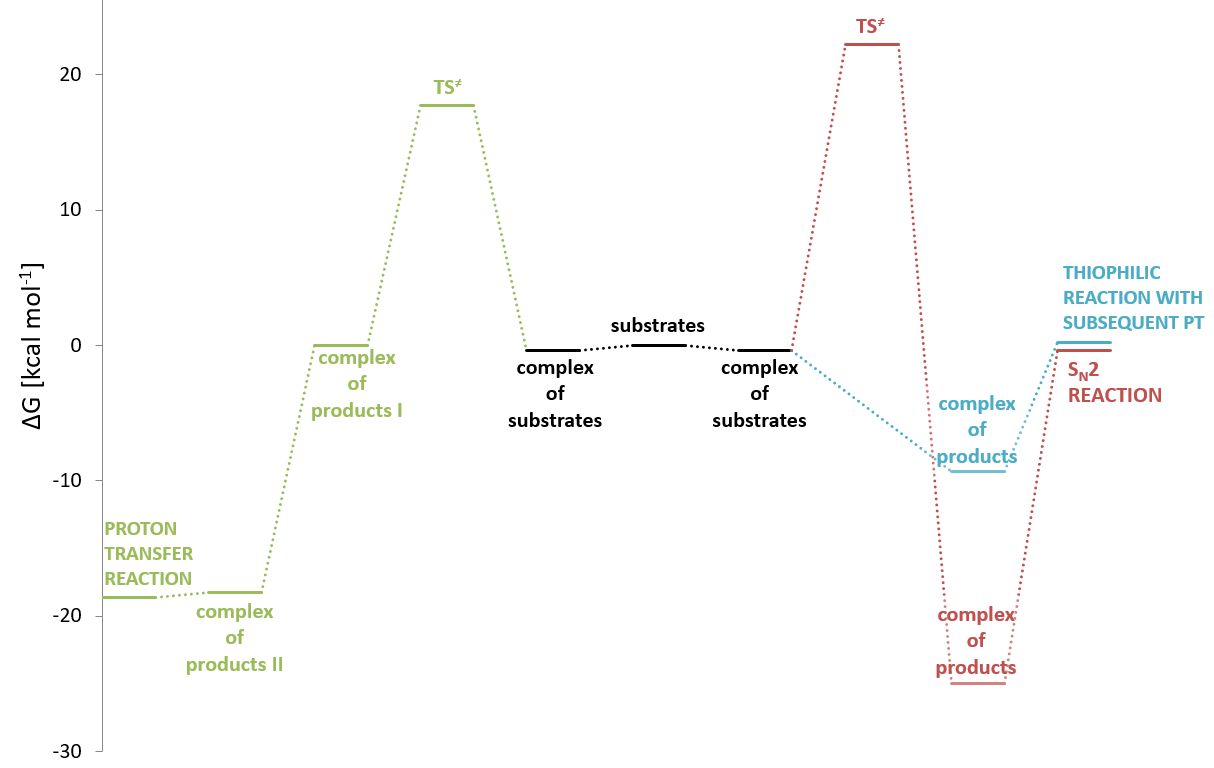


Figure S13. Gibbs free energy (Δ*G*) profiles of the reactions between ^-^CH_2_CO_2_Me and Me_2_S_2_


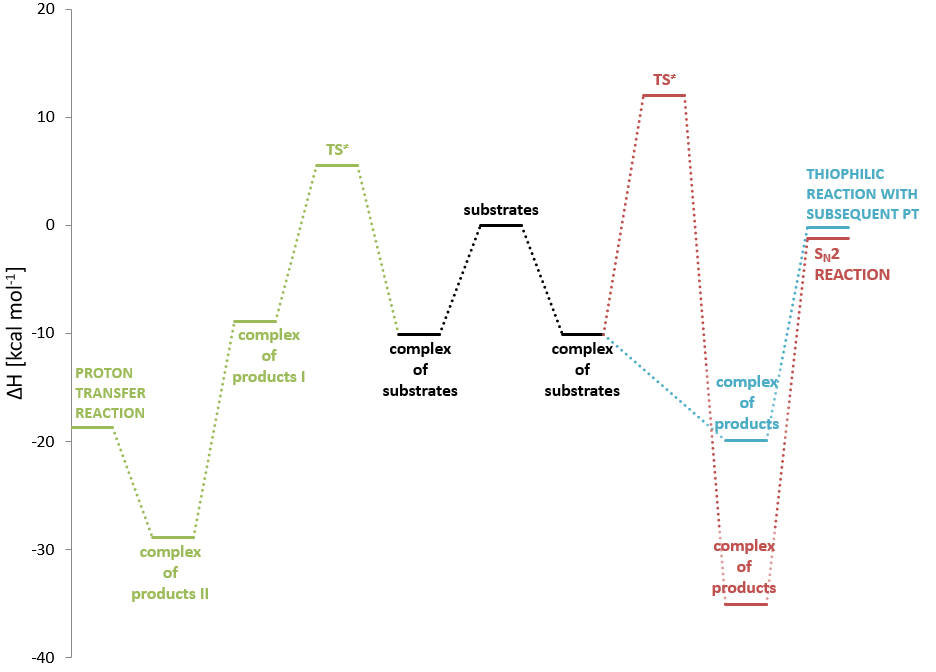


Figure S14. Enthalpy (Δ*H*) profiles of the reactions between ^-^CH_2_CO_2_Me and Me_2_S_2_


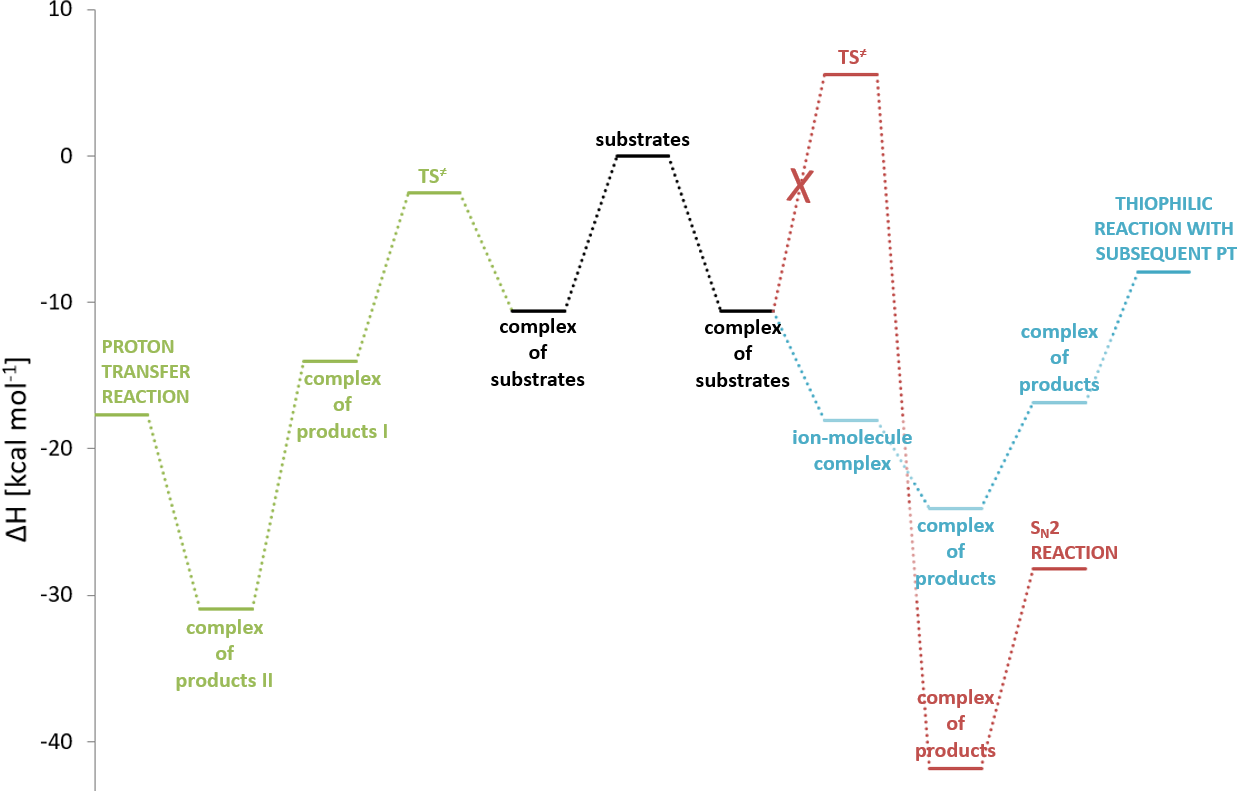


Figure S15. Enthalpy (Δ*H*) profiles of the reactions between ^-^CH_2_CN and Me_2_S_2_


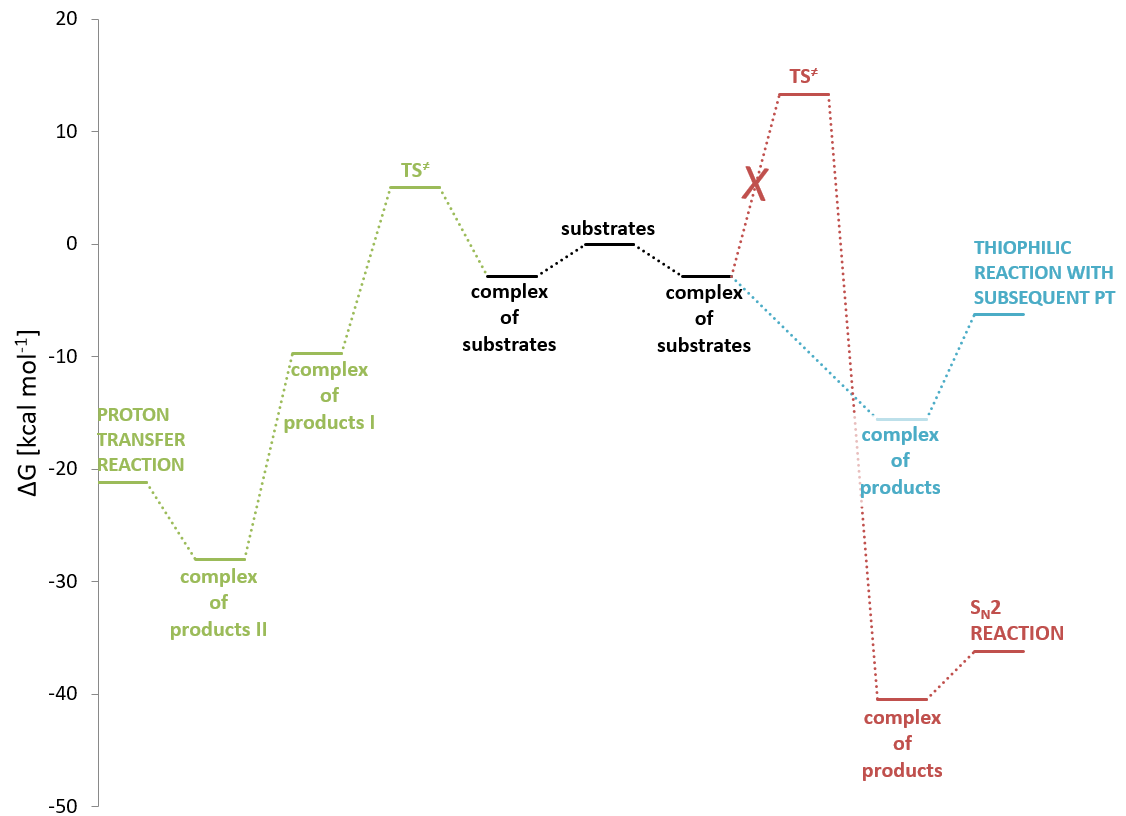


**Figure S16.** Gibbs free energy (Δ*G*) profiles of the reactions between ^-^CHCl_2_ and Me_2_S_2_


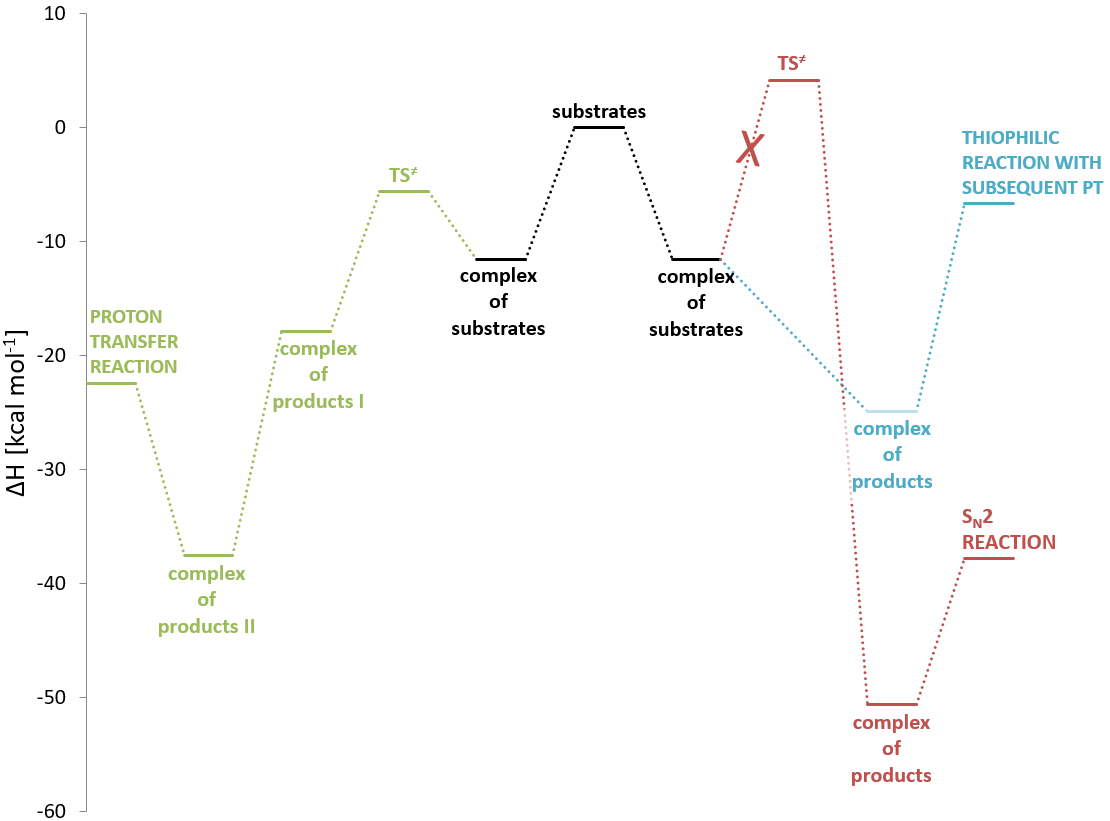


**Figure S17.** Enthalpy (Δ*H*) profiles of the reactions between ^-^CHCl_2_ and Me_2_S_2_


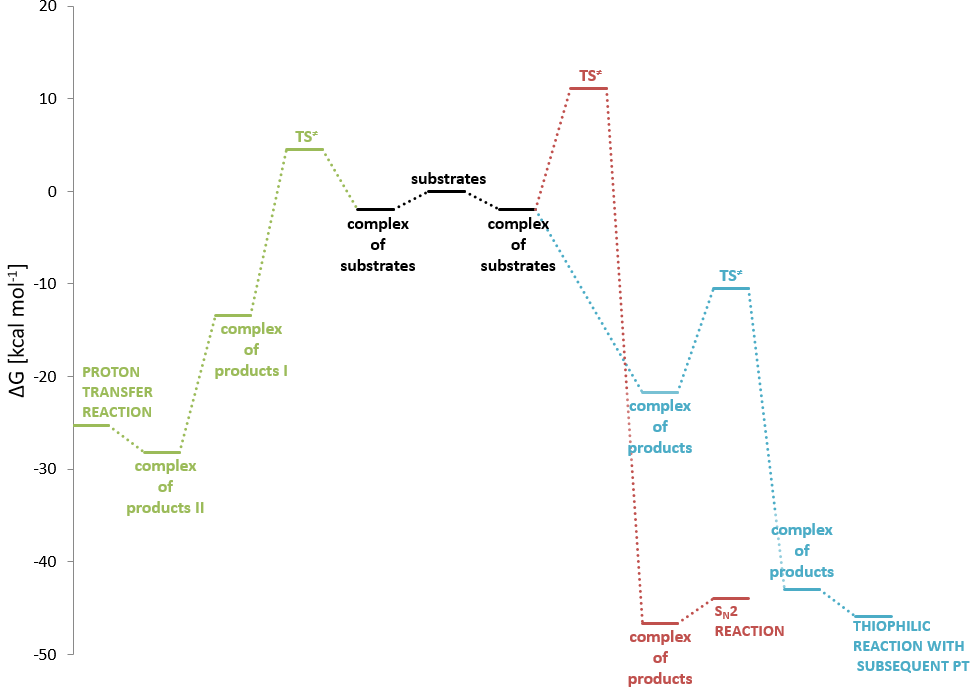


**Figure S18.** Gibbs free energy (Δ*G*) profiles of the reactions between ^–^CF_3_ and Me_2_S_2_


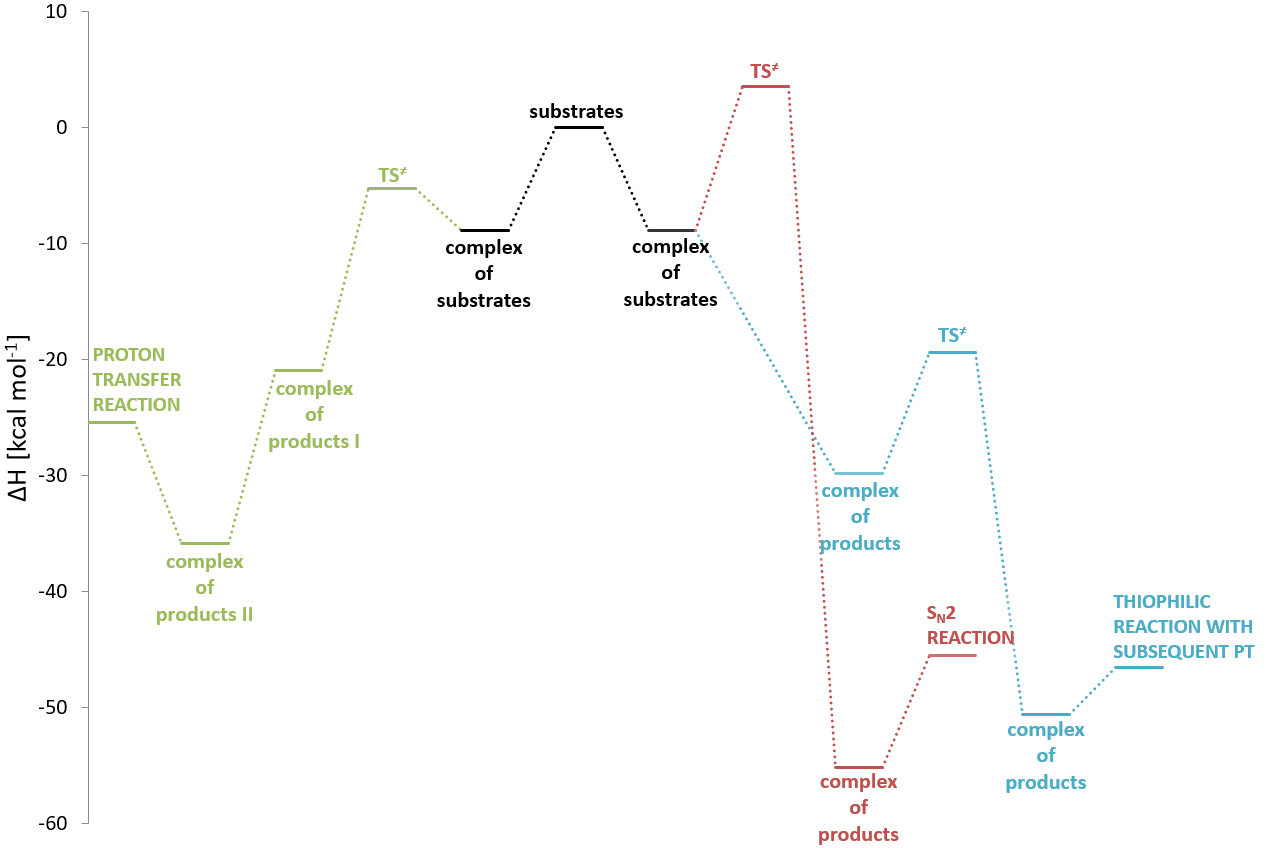


**Figure S19.** Enthalpy (Δ*H*) profiles of the reactions between ^–^CF_3_ and Me_2_S_2_


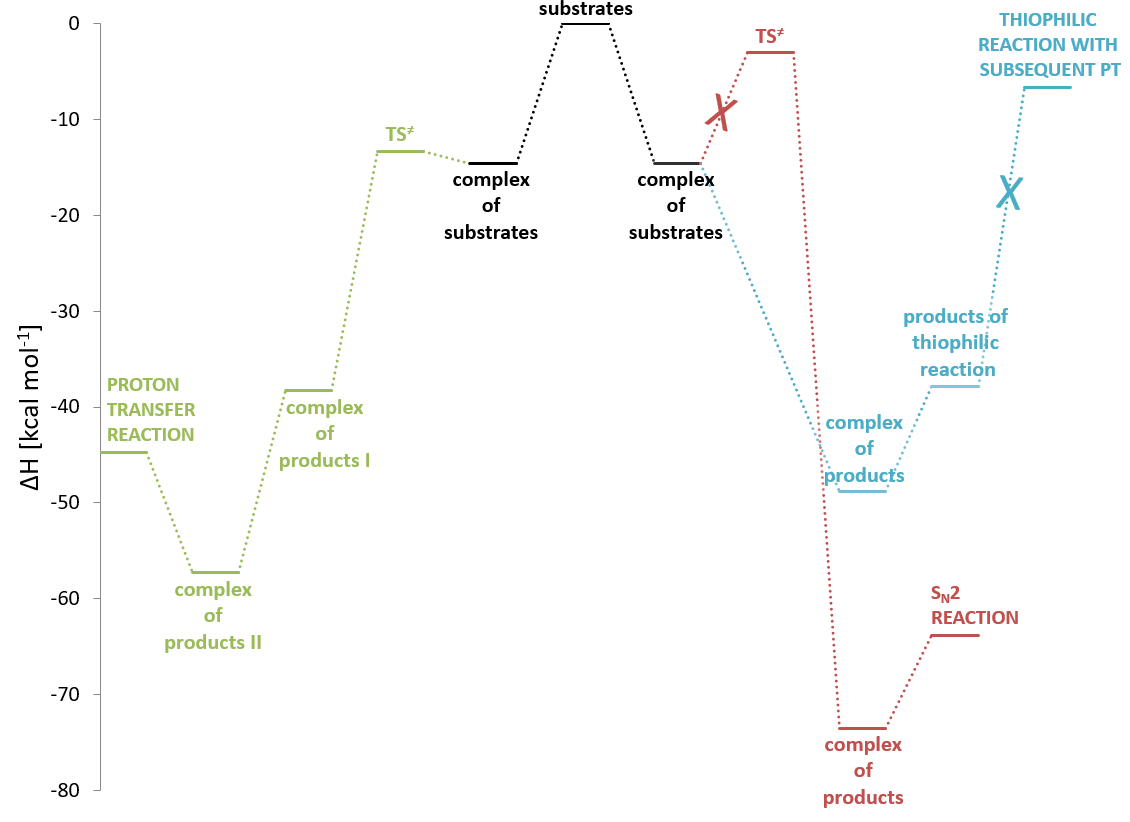


**Figure S20.** Enthalpy (Δ*H*) profiles of the reactions between ^–^CHF_2_ and Me_2_S_2_

**
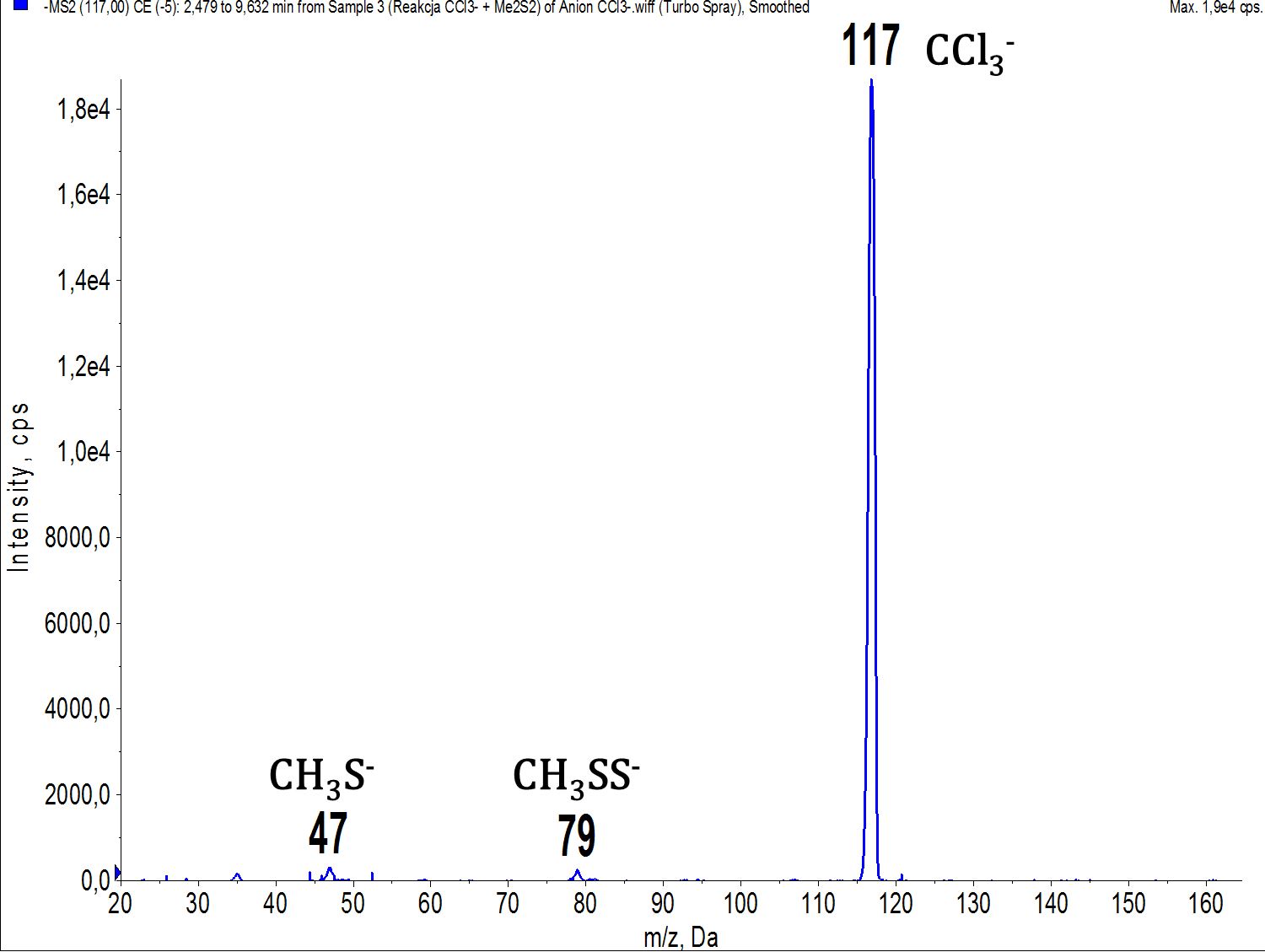

Figure S21**. Product ion spectrum recorded for the reaction of Me_2_S_2_ with ^–^CCl_3_


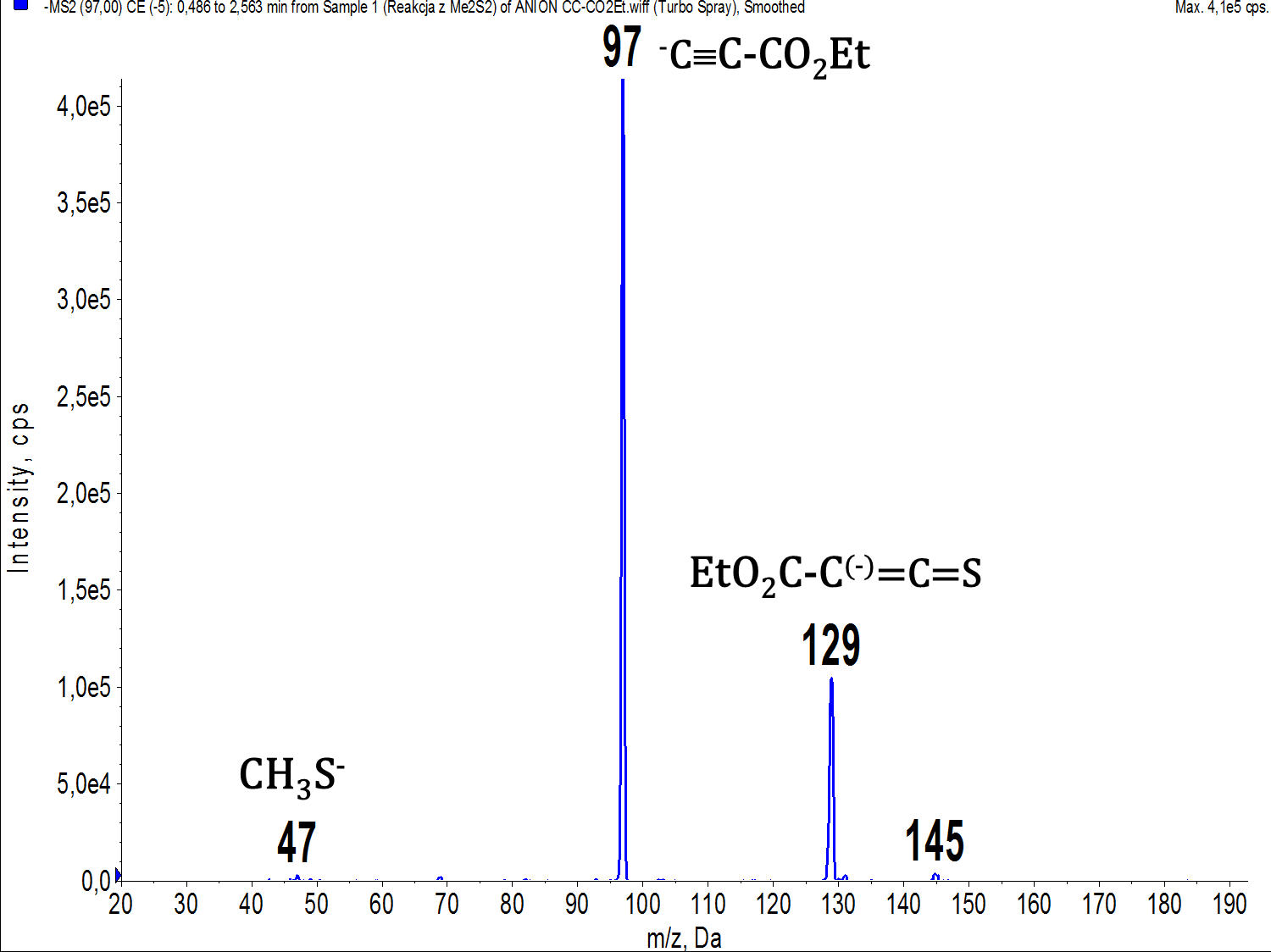


**Figure S22**. Product ion spectrum recorded for the reaction of Me_2_S_2_ with ^-^C≡C-CO_2_Et


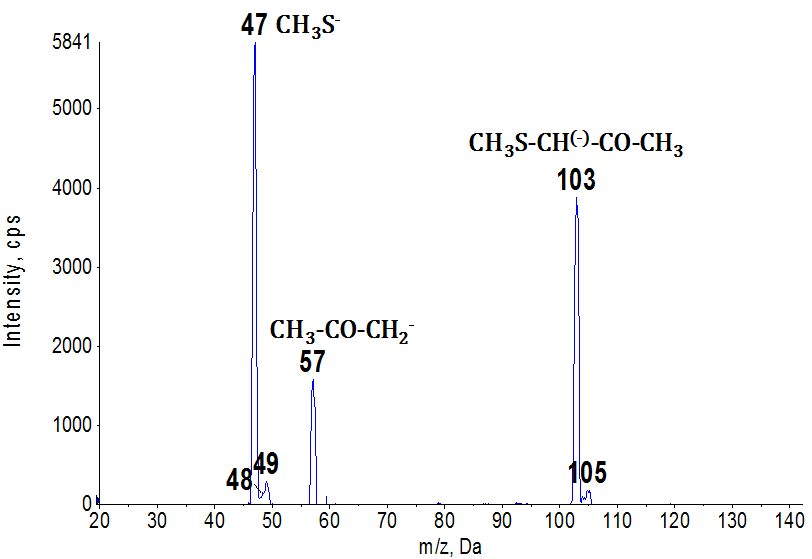


**Figure S23**. Product ion spectrum recorded for the reaction of Me_2_S_2_ with ^-^CH_2_-CO-CH_3_


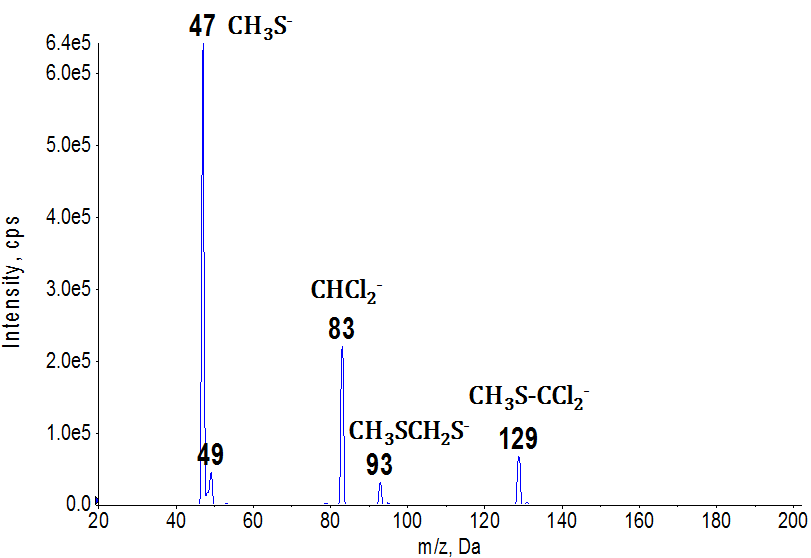


**Figure S24**. Product ion spectrum recorded for the reaction of Me_2_S_2_ with ^–^CHCl_2_


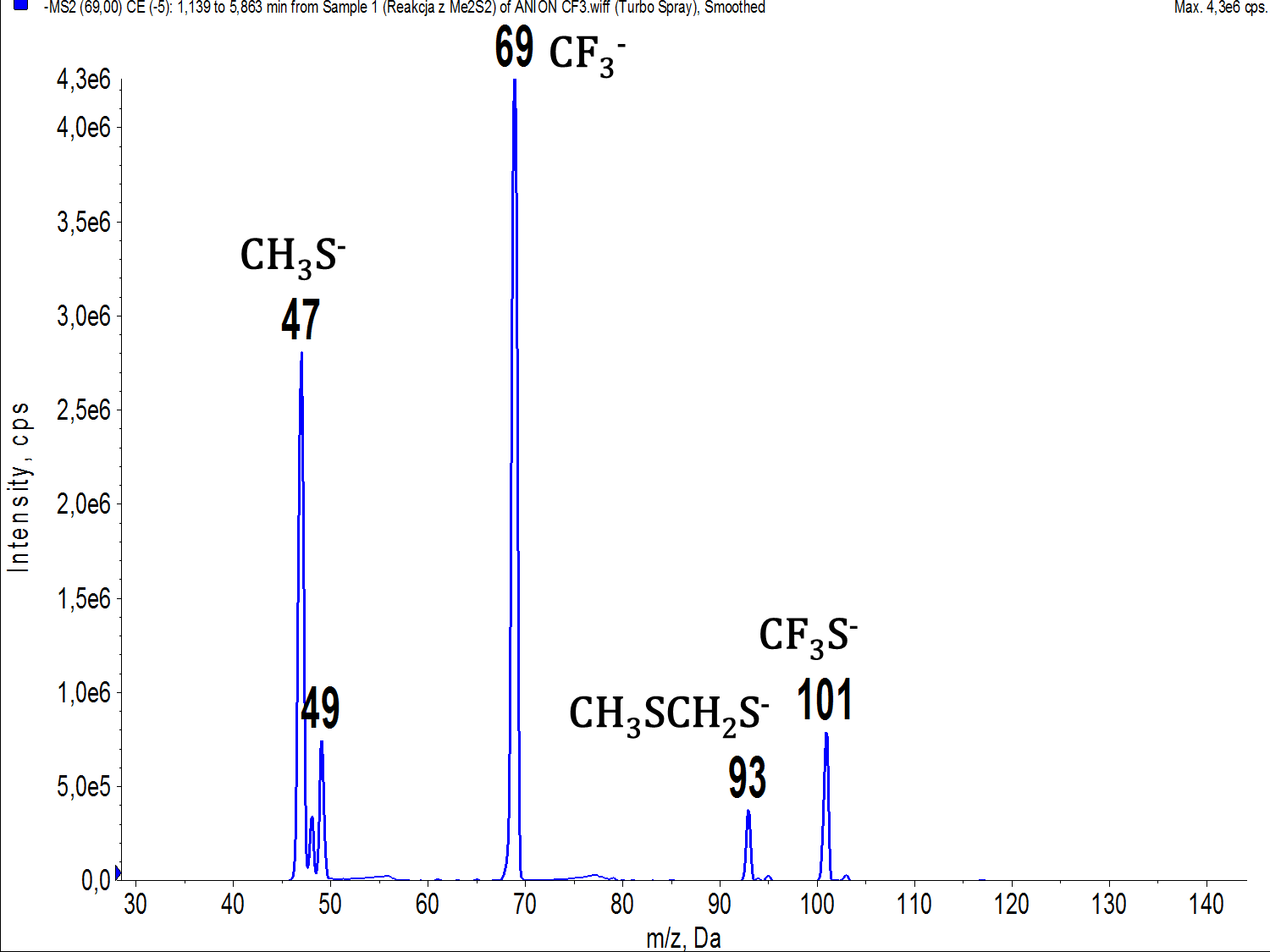


**Figure S25**. Product ion spectrum recorded for the reaction of Me_2_S_2_ with ^–^CF_3_
